# Angiotensin-Converting Enzyme (ACE) Inhibitors May Moderate COVID-19 Hyperinflammatory Response: An Observational Study with Deep Immunophenotyping

**DOI:** 10.34133/hds.0002

**Published:** 2022-12-27

**Authors:** Venkata R. Duvvuri, Andrew Baumgartner, Sevda Molani, Patricia V. Hernandez, Dan Yuan, Ryan T. Roper, Wanessa F. Matos, Max Robinson, Yapeng Su, Naeha Subramanian, Jason D. Goldman, James R. Heath, Jennifer J. Hadlock

**Affiliations:** 1 Institute for Systems Biology, Seattle, WA, USA.; 2 Department of Bioengineering, University of Washington, Seattle, WA, USA.; 3 Washington University, St. Louis, MO, USA.; 4 Swedish Center for Research and Innovation, Swedish Medical Center, Seattle, WA, USA.; 5 Providence St. Joseph Health, Renton, WA, USA.; 6 Division of Allergy and Infectious Diseases, University of Washington, Seattle, WA, USA.

## Abstract

**Background:**

Angiotensin-converting enzyme inhibitors (ACEi) and angiotensin-II receptor blockers (ARB), the most commonly prescribed antihypertensive medications, counter renin-angiotensin-aldosterone system (RAAS) activation via induction of angiotensin-converting enzyme 2 (ACE2) expression. Considering that ACE2 is the functional receptor for SARS-CoV-2 entry into host cells, the association of ACEi and ARB with COVID-19 outcomes needs thorough evaluation.

**Methods:**

We conducted retrospective analyses using both unmatched and propensity score (PS)-matched cohorts on electronic health records (EHRs) to assess the impact of RAAS inhibitors on the risk of receiving invasive mechanical ventilation (IMV) and 30-day mortality among hospitalized COVID-19 patients. Additionally, we investigated the immune cell gene expression profiles of hospitalized COVID-19 patients with prior use of antihypertensive treatments from an observational prospective cohort.

**Results:**

The retrospective analysis revealed that there was no increased risk associated with either ACEi or ARB use. In fact, the use of ACEi showed decreased risk for mortality. Survival analyses using PS-matched cohorts suggested no significant relationship between RAAS inhibitors with a hospital stay and in-hospital mortality compared to non-RAAS medications and patients not on antihypertensive medications. From the analysis of gene expression profiles, we observed a noticeable up-regulation in the expression of 1L1R2 (an anti-inflammatory receptor) and RETN (an immunosuppressive marker) genes in monocytes among prior users of ACE inhibitors.

**Conclusion:**

Overall, the findings do not support the discontinuation of ACEi or ARB treatment and suggest that ACEi may moderate the COVID-19 hyperinflammatory response.

## Introduction

As of January 2022, COVID-19, caused by the novel severe acute respiratory syndrome coronavirus 2 (SARS-CoV-2) was responsible for 5.47 million deaths with its persistent spread globally [1]. SARS-CoV2 entry into host cells is initiated by the binding of receptor-binding domain of the pathogen’s spike protein to the host cell receptor, angiotensin-converting enzyme 2 (ACE2) [2]. Physiologically, ACE2 is a part of the renin-angiotensin-aldosterone system (RAAS). Vasoconstriction, the most well-described function of RAAS activation, is mediated by the binding of angiotensin II to angiotensin II receptor type 1 (AT1R). ACE2 plays a critical role in countering the vasoconstriction induced by RAAS by catalyzing the conversion of angiotensin II into an intermediate effector molecule, angiotensin 1–7, that instead promotes vasodilation and anti-inflammatory responses [3,4]. RAAS inhibiting agents, including ACE inhibitors (ACEi) and angiotensin-II receptor blockers (ARBs), are commonly used treatment drugs for patients with hypertension and associated conditions. However, RAAS inhibiting agents might also facilitate the spread of SARS-CoV-2 by increasing expression of ACE2 [5,6] thus raising the concerns over the continuous use of ACEi and ARB treatment in the context of COVID-19 [7]. Conversely, since the down-regulation of ACE2 subsequent to SARS-CoV-2 infection could attenuate the protective effects of ACE2, the continued usage of ACEi and ARBs with resultant increase in ACE2 expression may be beneficial in preventing excessive activation of RAAS that may ensue following COVID-19 [5,8]. Given that the predominant response of AT1R activation is proinflammatory, RAAS activation on immune cells serves to limit their proinflammatory responses [9].

Several observational studies from the globe reported their evaluations on the use of antihypertensive medications affecting the ACE2 expression in the RAAS pathway for the SAR-CoV-2 infection and severe clinical outcomes. The association of ACEi or ARB did not correspond consistently with SARS-CoV-2 infection and poorer outcomes of COVID-19 [10–18]. Since ACEi and ARBs inhibit RAAS activation in different ways [19,20], their effect on COVID-19 outcomes may be distinct.

These contrasting propositions on drug-induced ACE2 expression levels and the associated clinical outcomes led us to elucidate the relationship between current use of first-line antihypertensive treatments and 2 primary outcomes: invasive mechanical ventilation (IMV) and 30-day mortality using retrospective observational data from electronic health records (EHRs) of Providence St. Joseph Health (PSJH) multistate healthcare network. Next, we analyzed ISB/Swedish INCOV data [21] of 139 COVID-19 patients that comprises circulating immune cells and plasma multiomic profiles associated with the PSJH-EHRs to examine the immunomodulatory potential due to current use of antihypertensive medications in hospitalized COVID-19 patients. Overall, this study evaluated the significance of current use of antihypertensive medications among hospitalized patients with SARS-CoV-2 infection.

## Methods

We conducted 2 complementary studies: (a) a retrospective observational study using EHRs and (b) analysis of data from a prospective observational study with deep immunophenotyping.

### Retrospective observational study using EHRs

**Fig. 1.**
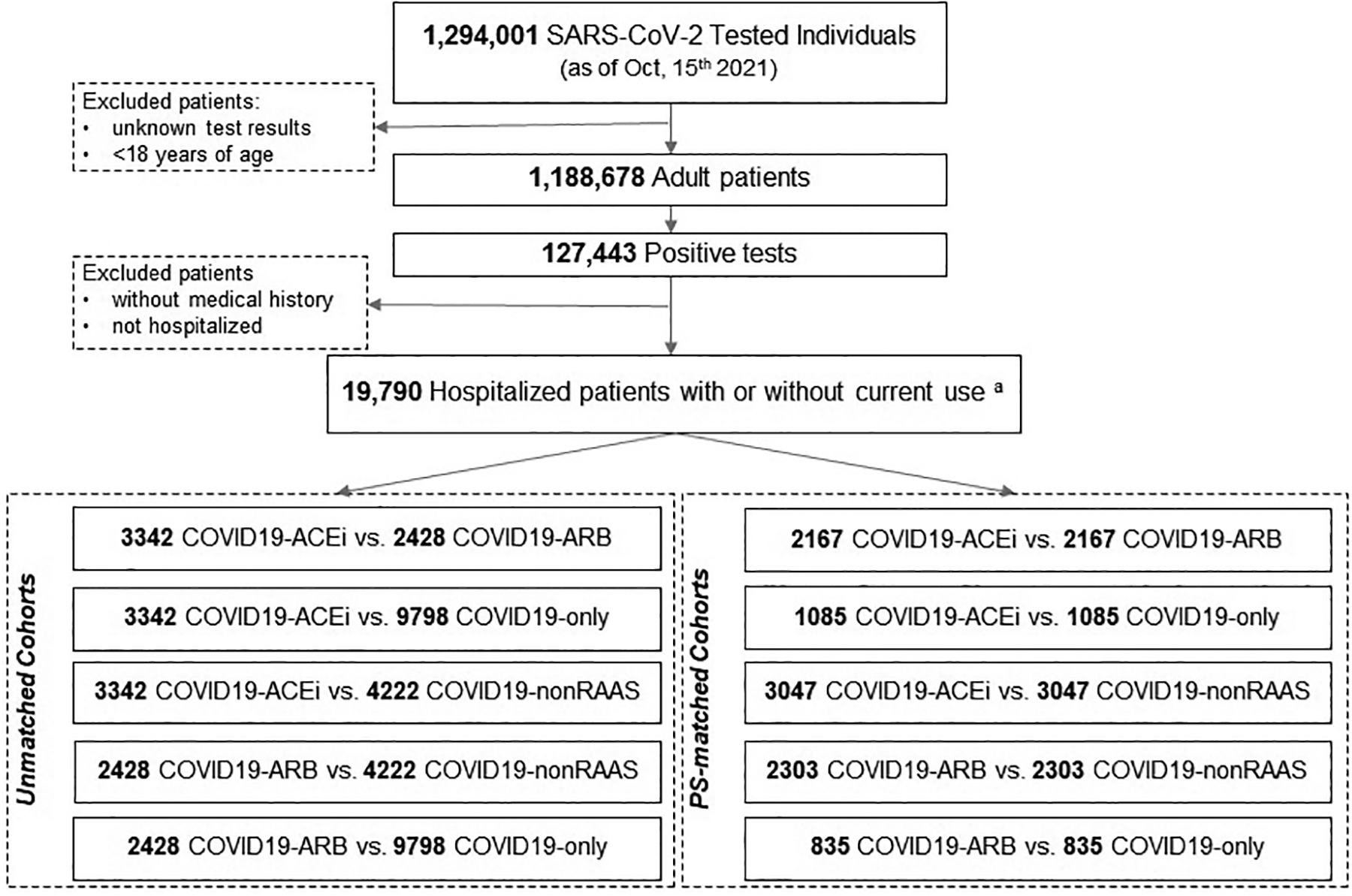
Study populations and cohort selection. Retrospective observational study: PS-matched cohort selection from PSJH-EHR data. Five cohorts were created based on patients’ use of antihypertensive drugs: COVID19-ACEi: Angiotensin-converting enzyme inhibitors; COVID19-ARB: Angiotensinogen-II receptor blocker; COVID19-nonRAAS: this group contains beta-blockers, calcium-channel blockers and diuretics; COVID19-only, COVID-19 patients without use of antihypertensive treatments based on their in-hospital medication history. Usage of antihypertensive drugs was defined based on the usage of indicated drugs a year prior to the index date or during in-hospital use. PJSH, Providence St. Joseph Health; PS-matched, propensity-score-matched; RAAS, Renin-angiotensin-aldosterone system; WOS, WHO Ordinal Scale for Clinical Improvement (Table S1).

EHR of hospitalized patients with COVID-19 were retrieved from PSJH, a multistate healthcare network that offers healthcare delivery in 5 states (Alaska, California, Montana, Oregon, and Washington) through community-based clinics and hospitals. Figure 1 presents the retrospective cohort flow diagram. Patients were excluded if they had no encounters at PSJH in the prior to COVID testing, to reduce bias from unknown medical and medication histories.

#### Study covariates and outcomes

The first contact date for a confirmed SARS-CoV-2 positive diagnosis test was used as the index date to define preexisting comorbidities and use of medications. We systematically evaluated EHRs to extract demographic, preexisting comorbidities, medication exposures, and disease severity. We used SNOMED CT (the Systematized Nomenclature of Medicine Clinical Terms) codes to extract preexisting comorbidities before the index date (Table S2). COVID-19 severity was determined for all confirmed SARS-COV-2-positive individuals by using EHR data to assign maximum World Health Organization (WHO) Ordinal Scale for Clinical Improvement (WOS) for each day of hospitalization (Table S1).

Patient drug exposures to the main classes of antihypertensive medications (ACEis, ARBs, beta-blockers, calcium-channel blockers, and diuretics) were gathered from the medication tables using RxNorm concept unique identifier (RxCUI) codes (Table S3). We defined antihypertensive drug exposure on the basis of usage of indicated drugs within the year prior to the index date or during in-hospital use. We then classified COVID-19 patients into 4 groups: (a) users of ACEis (COVID19-ACEi); (b) users of ARB (COVID19-ARB); (c) users of beta-blockers, calcium-channel blockers, and diuretics (COVID19-nonRAAS); and (d) COVID-19 patients without use of any antihypertensive drugs based on medication histories (COVID19-only). Combination therapy patients (~0.7%; *n* = 82) were excluded from this analysis. We selected covariates to include chronic conditions for which antihypertensive drugs are indicated, reported risk factors for severe COVID-19 outcomes (including age, sex, and chronic comorbidities), and medications used to reduce risk of hospitalization and death from chronic comorbidities. We also collected the antidiabetics, and lipid-lowering drugs, and anticoagulants with the similar definition of drug exposure. The study has the following outcomes: Comparison of time-to-IMV and comparison of time-to-death by day 30 on the 8-category WOS (Table S1).

#### Propensity score matching for balanced cohorts

Table presents unmatched treatment cohorts where significant *P* values indicate the imbalanced nature of each covariate, which could contribute to the differential response of treatment exposures against COVID-19. To improve the balance of covariates and reduce the effect of selection bias and potential confounding, we performed propensity score (PS)-matched analysis with the nearest-neighbor matching using MatchIt v4.0.0 package in R. PS-matched cohorts were created by matching all covariates listed through selection of patients from unmatched cohorts (Table). A greedy nearest-neighbor algorithm was used to match treated and control groups with a caliper distance (i.e., a maximum allowable difference in PS between treated and control groups) set to 0.01, resulting in a relatively narrow difference between matched subjects [22]. The balance of covariates was evaluated by estimating the standard mean difference (SMD) in PS, $\text{SMD}=\left({\bar{\text{x}}}_{\text{treatment}}-{\bar{\text{x}}}_{\text{control}}\right)/\surd {\text{s}}^{2}{}_{\text{treatmen}}+{\text{s}}^{2}{}_{\text{control}}/2$, before and after matching. The value of absolute SMD ≤ 0.1 was considered as an indicator of successful balancing between 2 groups [22]. SMD from the PS-matched analysis revealed the presence of covariate imbalance between unmatched groups with overall distance range from 0.475 to 1.243 (Fig. S1). Covariate imbalance was corrected (overall distance <0.001) for demographics, preexisting conditions, and medications to generate PS-matched cohorts (Table S4).

#### Statistical analysis

All categorical variables were presented as numbers, and percentages and continuous variables were presented as mean or median, standard deviation, and interquartile range. The statistical significance was calculated using Pearson’s Chi-square test (for categorical variables) and Kruskal-Wallis rank-sum test (for continuous variables). We used Kaplan-Meier (KM) survival analysis and log-rank test to compare the cumulative incidence of the length of hospital stay and survival among treatment groups [23]. Cox proportional hazard (CPH) regression analysis was performed to test the association between variables and outcomes. Visual assessment of KM curves to assess the proportional hazards assumption. Results are reported as point estimates (adjusted hazard ratio [aHR]) and 95% confidence intervals (95% CI) with *P* < 0.05 as statistically significant. R packages, survival v3.2.7 and survminer v0.4.8, were used to conduct KM and CPH analyses.

### Prospective observational study with deep immunophenotyping

#### Study subjects, WOS score, and EHR data extraction

The detailed methodology on collecting immune cells and plasma multi-omics of 139 patients has been reported by Su et al. [21]. Briefly, patients with SARS-CoV-2 infection diagnosed by polymerase chain reaction were enrolled into cohort study when presenting for care. The first blood draw (T1) after study enrollment was used for this analysis, which was typically collected in the first week after COVID-19 diagnosis / of infection (median d 0.67 [interquartile range 0.21], range: −1.75 to 4.54 d). Whole blood was processed within 4 h to peripheral blood mononuclear cells and plasma. Thawed peripheral blood mononuclear cells were >95% viable and were used in single-cell RNA sequencing (10× Genomics) experiments. Circulating immune cell phenotypes and gene expression data were determined as previously described [21]. The use of antihypertensive treatment and clinical lab results were extracted from EHR. Twenty-seven EHR clinical laboratory markers (complete blood count [CBC] with differential, metabolic panel, coagulation panel, and SPO2) were collected from the nearest time point to T1 if available within a window ± 2 d. Seventy-three hospitalized patients (with WOS 3 to 8) of 139 INCOV study patients were classified into 4 groups (as described earlier) on the basis of their exposures to the antihypertensive medications (Fig. S2). Sixteen healthy donors were included in this study to compare circulating immune cell subtype percentage distributions and gene expressions.

#### Gene selection and differential expression

Identification of statistically significant differences in gene expression per cell type between any 2 of our treatment groups required a significant filtering process prior to any statistical analysis. We first calculated the difference in means $\left({\bar{\text{x}}}_{\text{group A}}-{\bar{\text{x}}}_{\text{group B}}\right)$ for each gene to identify genes which potentially drive significant changes between the cohorts and ranked them by their absolute value. We then performed a Wilcoxon rank-sum test on the top 150 genes to test whether these differences are significant. From there, we controlled for the resulting false discovery rate (FDR) using the algorithm laid out in remark B of Storey and Tibshirani [24], with a conservative modification of interpolating to 0.01 beyond the largest *P* value present, as opposed to interpolating all the way to 1. Once the positive FDR *q* values have been calculated, we further filter the results by choosing genes with *P* < 0.05 and *q* < 0.01. Finally, we filtered once more by picking genes with fold change >2 and fold change <0.5 and used this procedure for comparisons between 5 groups, COVID19-ACEi, COVID19-ARB, COVID19-nonRAAS, COVID19-only and Healthy donors, as well as across 5 immune cell types, B cells, CD4^+^ T cells, CD8^+^ T cells, monocytes, and natural killer (NK) cells.

#### Unsupervised learning

Hierarchical clustering was performed to compare clinical profiles of hospitalized COVID-19 patients with or without antihypertensive medication. We measured the (dis)similarity of covariates using Euclidean distance metric and dissimilarity between 2 clusters using the Ward.D linkage method. Python package scikit-learn 0.24.2 was used. We included clinical labs, vitals, preexisting conditions, gene expression, vitals, disease severity score, and demographics in clustering analysis. Missing labs were assumed to be normal given missingness was generally only for outpatients who were asymptomatic or had only mild symptoms with COVID-19. The median values of the normal lab reference range for adults from American Board of Internal Medicine were used to impute missing labs. Outliers were not removed, given that results had been reviewed during clinical chart abstraction and results outside of reference range contain relevant signal for disease states.

#### Supervised machine learning

Ensemble-based machine learning algorithms, random forest (RF), Adaptive Boosting (AdaBoost), gradient boosting (GB) and extreme gradient boosting (XGBoost) were implemented to find out the variables that have significant association with treatment groups. Python packages scikit-learn 0.24.2 (sklearn. ensemble module for RF, AdaBoost, and GB) and xgboost 1.4.1 were utilized. In this analysis, COVID19-ACEi and COVID19-ARB patients were grouped as COVID19-RAASi (renin-angiotensin-aldosterone system inhibitors), and modeling was conducted in comparison with COVID19-nonRAAS, and COVID19-only, separately. Classifier models were trained to discriminate COVID19-RAASi from COVID19-nonRAAS and COVID19-only. Ten-fold cross-validation procedure was used to minimize overfitting. Evaluation metrics such as accuracy, precision, recall, F1-score, and area under the receiver operating characteristic curve (AUROC) were measured to assess each model performance. The Shapley additive explanations (SHAP, v0.37.0) [25] package was used to identify the top discriminatory covariates from each model, and model interpretability was established via SHAP plots. SHAP assigns each feature an importance value (the Shapley value) for each specific classification.

## Results

### Retrospective observational study using electronic health records

#### Baseline characteristics of antihypertensive treatment groups

Within the PSJH network, 1,188,678 adults (age ≥ 18 years) underwent SARS-CoV-2 testing as of 2021 October 15. Of those tested, 127,443 (10.72%) were diagnosed with SARS-CoV-2 infection, confirmed by polymerase chain reaction. Of those infected, 19,790 (15.5%) were hospitalized, with a mean age of 63.11 ± 17.68 years. COVID19 patients were classified by antihypertensive use, either none or by type (ARB, ACEi, or non-RAAS); characteristics of these 4 patient groups are reported in Table. Prior to PS matching, the mean ages for subcohorts were 68.36 ± 14.48 for COVID19-ACEi, 71.77 ± 13.47 for COVID19-ARB, 66.52 ± 17.02 for COVID19-nonRAAS, and 57.20 ± 18.21 for COVID19-only.

These characteristics provide both similarities and differences among the 4 antihypertensive administration classes. In all groups, males outnumbered females. Most of the preexisting comorbidities, including hypertension, type 2 diabetes, obesity, chronic kidney disease, heart failure, and coronary artery disease, were more than twice as common among patients given antihypertensives than those not, and significantly more common when the administered antihypertensive was ACEi or ARB than a non-RAAS drug. Heparin was administered to most hospitalized COVID-19 patients (63.7%); either heparin or another anticoagulant was more likely to be administered to patients also receiving an antihypertensive. Antidiabetic and lipid-lowering drugs were also more commonly administered to patients receiving antihypertensive drugs, and more commonly administered along with ACEi or ARB than with a non-RAAS antihypertensive.

COVID-19 patients receiving antihypertensive drugs had significantly fewer mild and significantly more severe hospitalizations, with or without IMV, than patients not on antihypertensives (χ^2^ = 228.1, df = 3, *P* = 3.5 × 10^−49^), but also a slightly lower death rate (14.1% vs. 15.0%; see Table S5). Comparison of the different antihypertensive drugs revealed a significant and consistent trend of outcome severities (COVID19-ACEi < COVID19-ARB < COVID19-nonRAAS; χ^2^ = 183.9, df = 6, *P* = 5.1 × 10^−37^), with similar rates of severe hospitalization without IMV but more mild hospitalizations, fewer severe hospitalizations with IMV and lower rates of death for COVID19-ACEi than COVID19-ARB than COVID19-nonRAAS antihypertensives, despite the opposite trend for comorbidities and concurrent medications (Table).

This descriptive analysis from the unmatched cohorts (Table) revealed that being male, having a high disease burden and being on antidiabetic, anticoagulant, and lipid-lowering prescription medications are likely specific characteristics of hospitalized COVID-19 patients taking antihypertensive medications. Additionally, COVID19-only were observed to have lower rates of IMV than COVID-19 patients on any of antihypertensive medications. Overall, unmatched cohorts revealed the significantly imbalanced nature of covariates between treatment groups. Hence, to avoid the effect of this imbalance on outcomes, we implemented PS matching. Results are reported from both unmatched cohorts (Table) and PS-matched cohorts (Table S4).

#### Association of ACEi or ARBs with risk of receiving IMV in hospitalized COVID-19 patients

**Fig. 2.**
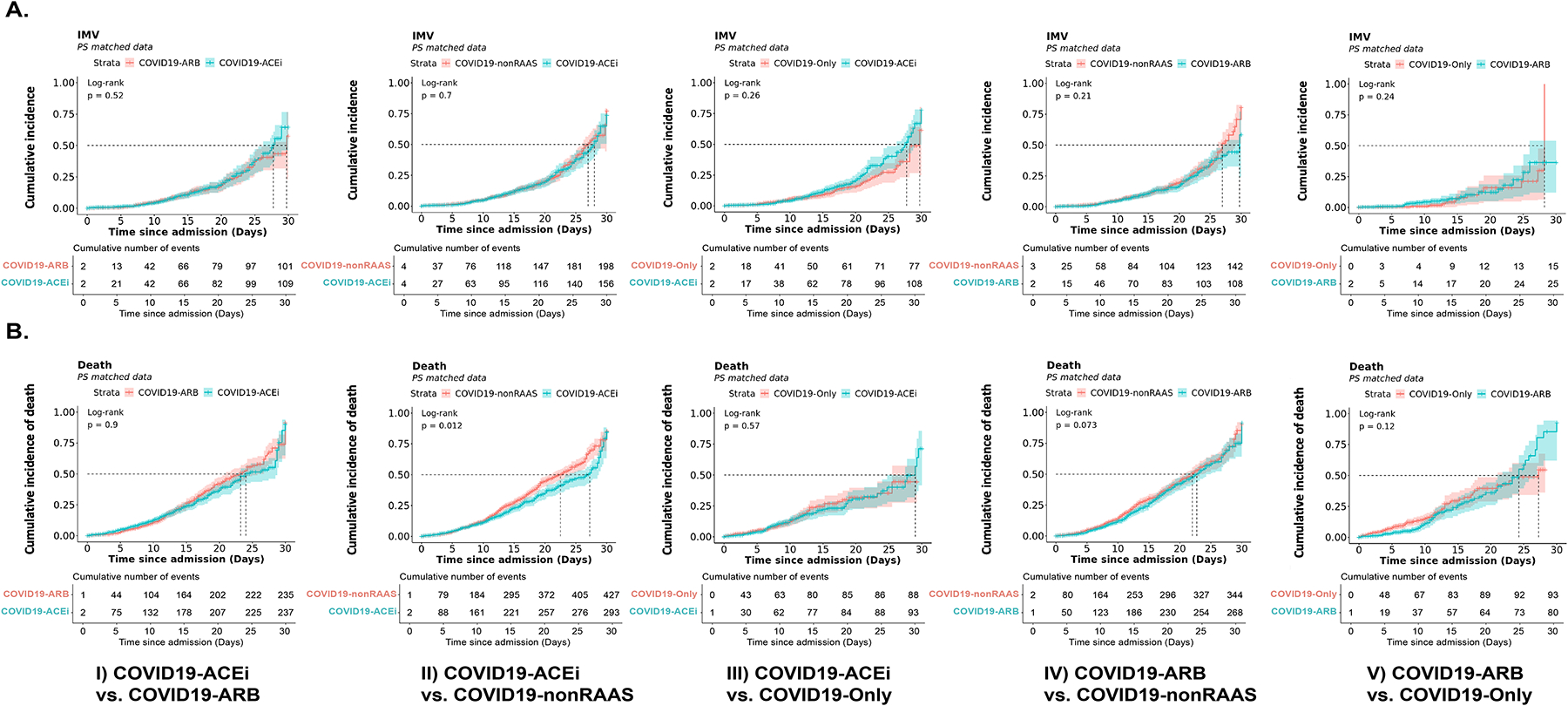
KM survival curves using PS-matched data of hospitalized patients with COVID-19 with or without use of antihypertensive drugs. (A) Survival curves for receiving invasive mechanical ventilation (IMV) or ECMO (WOS, 6, 7). (B) Survival curves for mortality (WOS, 8). Survival analysis was conducted using 5 PS-matched cohorts: COVID19-ACEi vs. COVID19-ARB; COVID19-ACEi vs. COVID19-nonRAAS; COVID19-ACEi vs. COVID19-only; COVID19-ARB vs. COVID19-nonRAAS and COVID19-ARB vs. COVID19-only. Log-rank *P* values are for the difference between treatments where *P* < 0.05 considered to be significantly different. Numbers provided indicate the cumulative number of events in each cohort. Table S4 presents characteristics of PS-matched cohorts.

KM survival analyses with PS-matched cohorts (Table S4 and Fig. 2A) revealed no significant risk associated with receiving IMV in either treatment group (Fig. 2A). KM survival analysis with unmatched cohorts (Table and Fig. S3A) did show increased risk of receiving IMV among users of ACEis (COVID19-ACEi) compared to the COVID19-only group (HR, 1.21, 95% CI [1.01, 1.46], log-rank *P* = 0.038) (Fig. S3A, column 3).

**Fig. 3.**
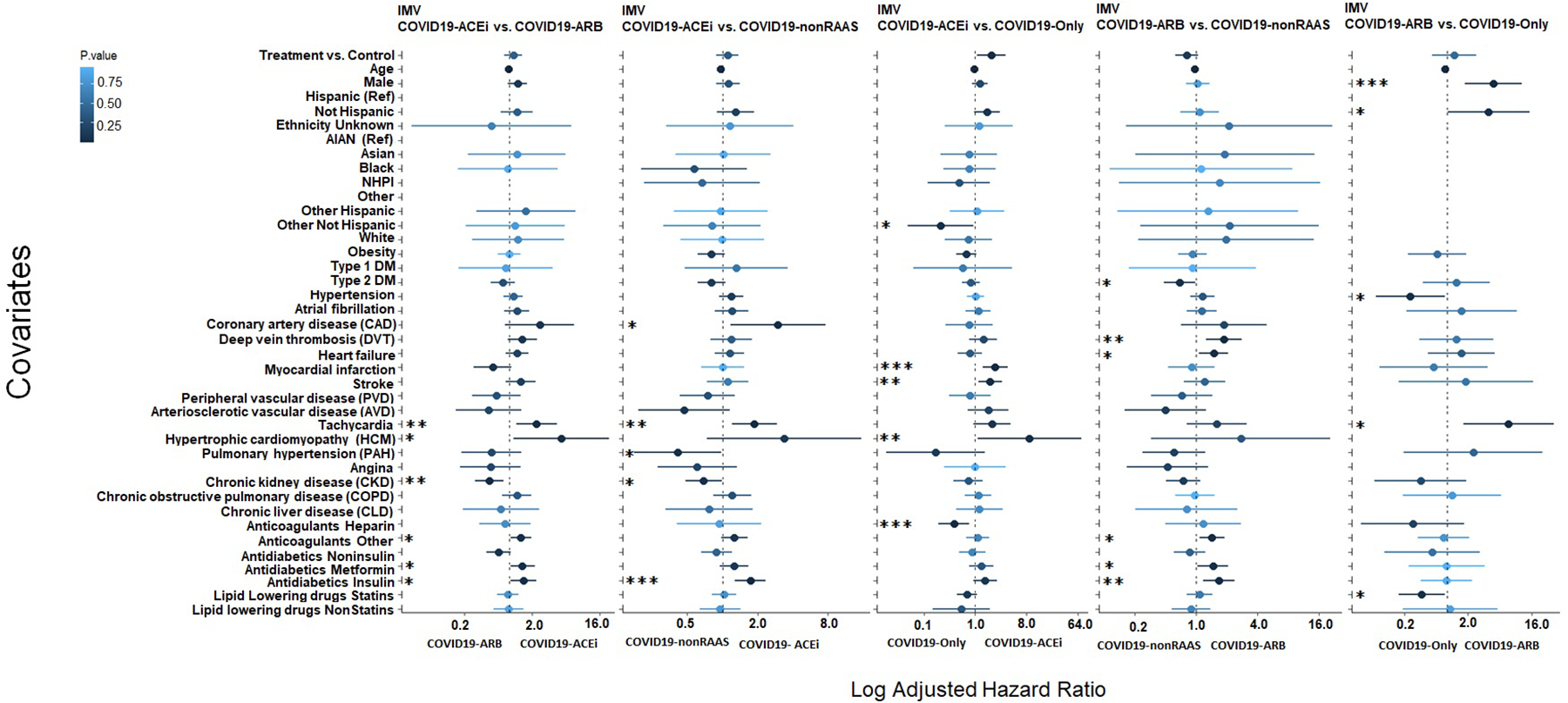
CPH regression analysis: Forest plot subgroup analysis with PS-matched cohort to identify covariates that differentiate treatment groups for risk of receiving IMV (WOS = 6 or 7). The solid dots represent the log adjusted hazard ratio (HR), the horizontal lines associated with each dot represents the 95% confidence interval (CI), and the color of dot indicates the level of significance as denoted by the color bar. In addition, p-significance of each covariate is represented with asterisk(s), *P < 0.05, **P < 0.01, ***P < 0.001. The dashed vertical line denotes the line of null effect. Table S4 presents characteristics of PS-matched cohorts. WOS, WHO ordinal scale (Table S1).

CPH analysis (with PS-matched cohorts) adjusted for all covariates indicate that use of ACEis (COVID19-ACEi) was significantly associated with the increased risk of receiving IMV (aHR, 1.91; 95% CI [1.07, 3.4]; *P* = 0.027) compared to the COVID19-only (Fig. 3, column 3). The covariates, preexisting myocardial infarction (aHR, 2.24; 95% CI [1.34, 3.71]; *P* = 0.001), hypertrophic cardiomyopathy (HCM) (aHR, 8.99; 95% CI [1.11, 72.45]; *P* = 0.03) and stroke (aHR, 1.83; 95% CI [1.12, 2.99]; *P* = 0.01) were associated with the increased risk of receiving IMV among hospitalized COVID19-ACEi group. The race other (aHR, 0.24; 95% CI [0.06, 0. 29]; *P* = 0.03) and use of anticoagulant heparin (aHR, 0.42; 95% CI [0.22, 0.78]; *P* = 0.01) was associated with reduced risk of receiving IMV in the hospitalized COVID19-only group. No significant association of treatment was observed in other comparative treatment groups with PS-matched cohorts (Fig. 3).

#### Association of ACEi or ARBs with risk of mortality in hospitalized COVID-19 patients

**Fig. 4.**
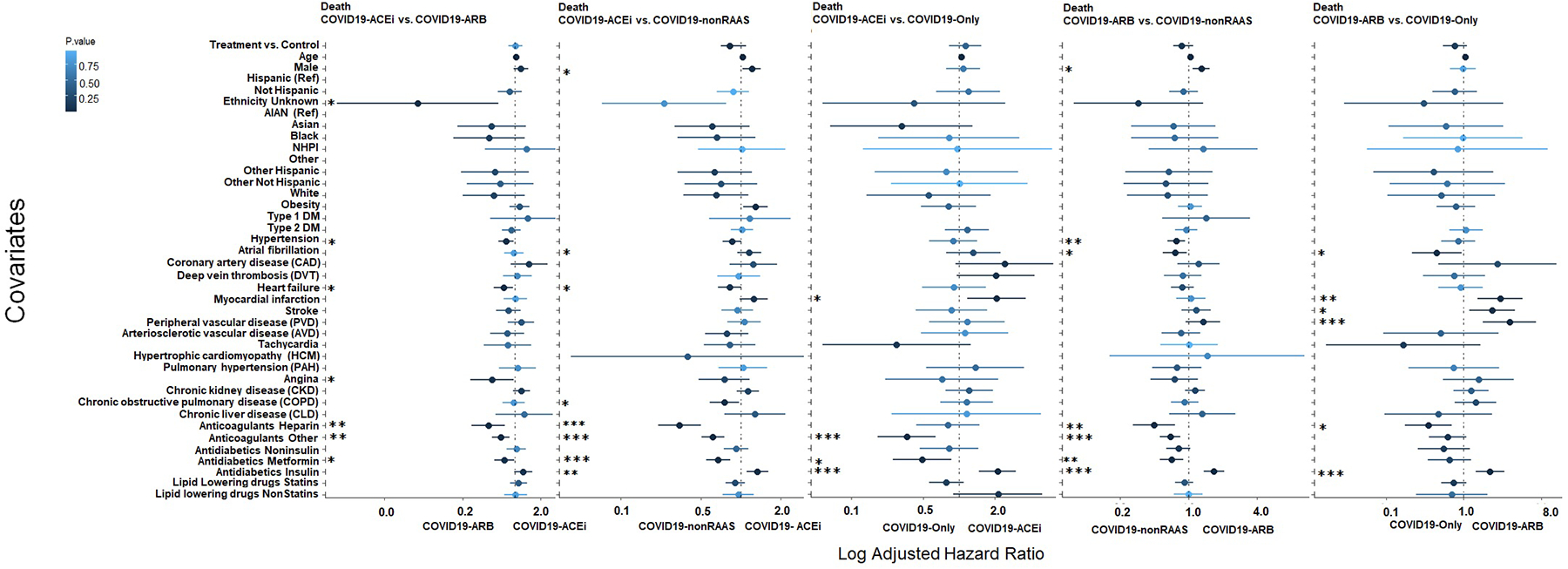
CPH regression analysis: Forest plot subgroup analysis with PS-matched cohort to identify covariates that differentiate treatment groups for risk of mortality (WOS = 8). The solid dots represent the log adjusted hazard ratio (HR), the horizontal lines associated with each dot represent 95% confidence interval (CI), and the color of dot indicates the level of significance as denoted by the color bar. In addition, p-significance of each covariate is represented with asterisk(s), *P < 0.05, **P < 0.01, ***P < 0.001. The dashed vertical line denotes the line of null effect. Table S4 presents characteristics of PS-matched cohorts. WOS, WHO ordinal scale (Table S1).

KM survival analyses with PS-matched cohorts (Table S4 and Fig. 2B) revealed that compared to use of nonRAAS, use of ACEi was associated with reduced risk of mortality (HR, 0.82; 95% CI [0.64, 0.92]; log-rank *P* = 0.01). None of the other treatment comparisons were significantly different with PS-matched cohorts (Fig. 2B). CPH analysis (with PS-matched cohorts) adjusted for all covariates indicated that use of non-RAAS (COVID19-nonRAAS) were not significantly associated with the reduced risk of mortality compared to the COVID19-ACEi. (Fig. 4, column 2). No significant association of treatment was observed in other comparative treatment groups with PS-matched cohorts (Fig. 4). With the unmatched cohorts (Table and Fig. 1), we noticed a trend of reduced risk of death among control groups compared to COVID19-ACEi and COVID19-ARB except COVID19-ARB vs. COVID19-nonRAAS analysis (Fig. S3B).

Together, comparative analysis with different combinations of treatment groups revealed that use of ACEis was marginally associated with risk of receiving IMV (*P* = 0.027) when compared to COVID-19 patients without use of any antihypertensive drugs based on medication histories (COVID19-only) but not with other comparisons including COVID19-ARB. Most of these findings are in agreement with findings from other studies [10–14] that have not observed highly significant risk association of ACEi or ARB usage on COVID-19 clinical outcomes and suggest that the usage of ACEi or ARB medications can be continued when indicated in patients with COVID-19 [10–14,18].

### Prospective observational study with deep immunophenotyping

RAAS blockade by ACEi or ARB therapy in hypertension patients has been previously shown to reduce systemic inflammation [26–29], so we analyzed circulating immune cell phenotypes and gene expression data to investigate potential immunomodulatory effects of ACEi or ARB therapies. Seventy-three patients were identified from the ISB/Swedish INCOV study [21] by reviewing use of antihypertensive medications.

#### No noticeable changes observed in the circulating immune cell subtypes in treatment groups

**Fig. 5.**
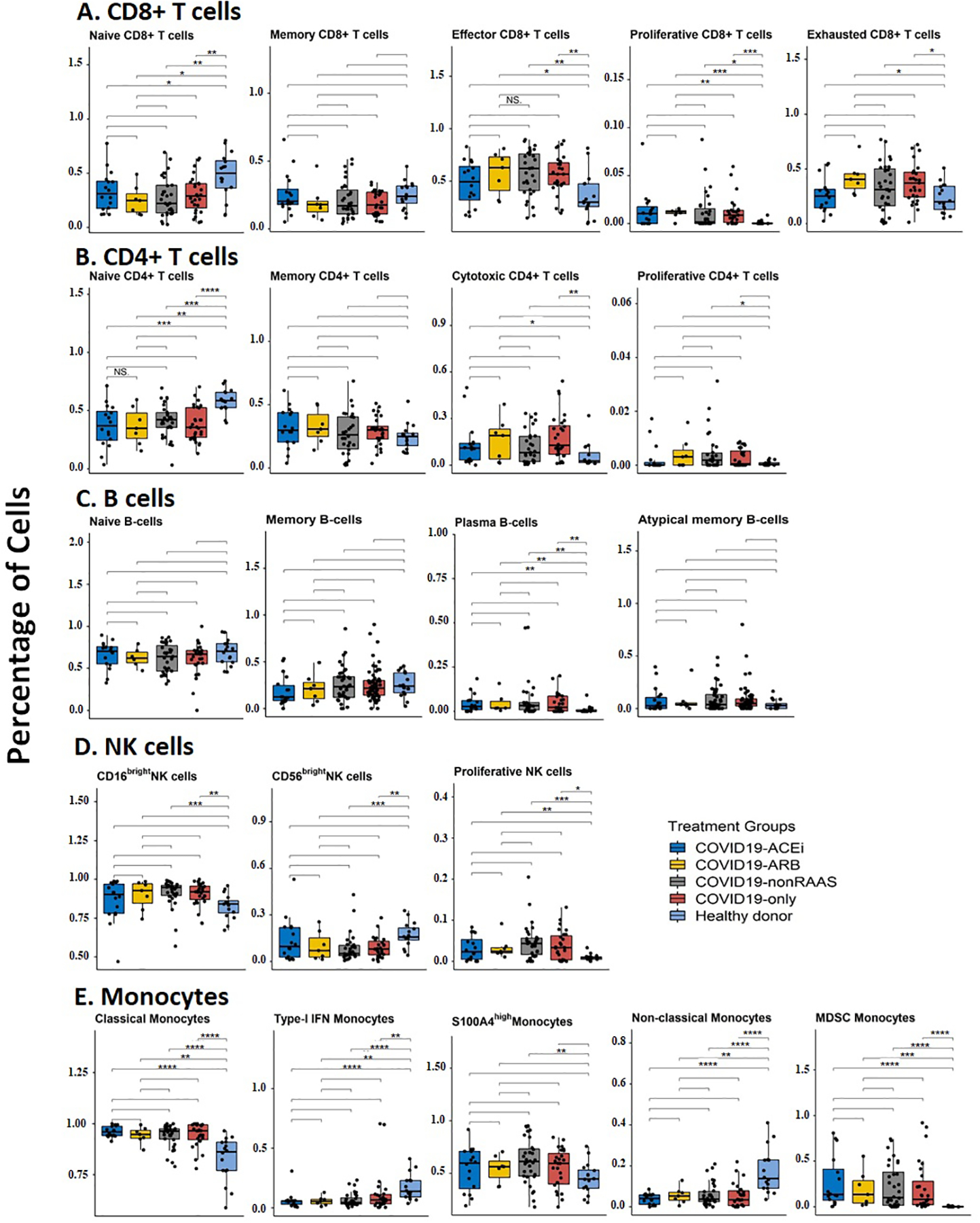
Circulating immune cell subtype percentage distribution among hospitalized COVID-19 patients with or without use of antihypertensive drugs. Statistical significance represented as Bonferroni-adjusted p-significance in symbols ns: *P* > 0.05, *P ≤ 0.05, **P ≤ 0.01, ***P ≤ 0.001, and ****P ≤ 0.0001. The nonsignificant symbols (ns) were hidden on all boxplots.

Irrespective of the antihypertensive treatment use, including ACEi or ARB, our cohort showed significantly (bonferroni corrected *P* significance) reduced levels of naïve CD8^+^ and CD4^+^ T cells (Fig. 5A and B) compared to healthy donors, which is consistent with the severe T cell depletion in COVID-19 patients [21,30]. However, it is notable that compared to healthy donors, COVID-19 patients with a use of ARB but not with ACEi or non-RAAS treatment, showed a higher percentage of exhausted CD8^+^ T cells (Fig. 5A). For CD4^+^ T cell subgroups, users of ACEi and nonusers of antihypertensive drugs (COVID19-only) showed significantly elevated levels of cytotoxic CD4^+^ T cells compared to healthy donors (Fig. 5B). Plasma B cell (Fig. 5C) and NK cell (Fig. 5D) populations were not found to be significantly different in antihypertensive treatment groups.

Although percentages of classical monocytes compared to healthy donors were significantly elevated in all patient groups, independent of antihypertensive drug usage, percentages of type-1 interferon producing monocytes were significantly reduced (Fig. 5E) in line with dysregulated interferon responses reported in COVID-19 patients [21,31]. Further, percentages of nonclassical monocytes known for their role in wound healing and resolution of inflammation [32] were significantly decreased in COVID-19 patients compared to healthy donors, and no differences in monocyte cell types were observed among patient groups based on antihypertensive drug usage (Fig. 5E). Considering that myeloid-derived suppressor cell (MDSC) monocytes preferentially expand in the conditions of pathological settings as a protective immunosuppressive mechanism [33], we next analyzed the frequency of circulating MDSCs in our cohorts. As shown in Fig. 5E, patients with COVID-19 have elevated levels of MDSC compared to healthy controls; however, there are no differences in MDSC frequencies among COVID-19 patients based on treatment. In summary, the COVID19-ACEi and COVID19-ARB patient groups did not exhibit significant differences in the distributions of circulating immune cell subtypes including the frequency of immunosuppressive MDSC compared to the COVID19-nonRAAS and COVID19-only groups.

#### Up-regulated anti-inflammatory and immune suppressive genes in ACEi users may moderate hyperinflammatory gene signature of COVID-19 infection

**Fig. 6.**
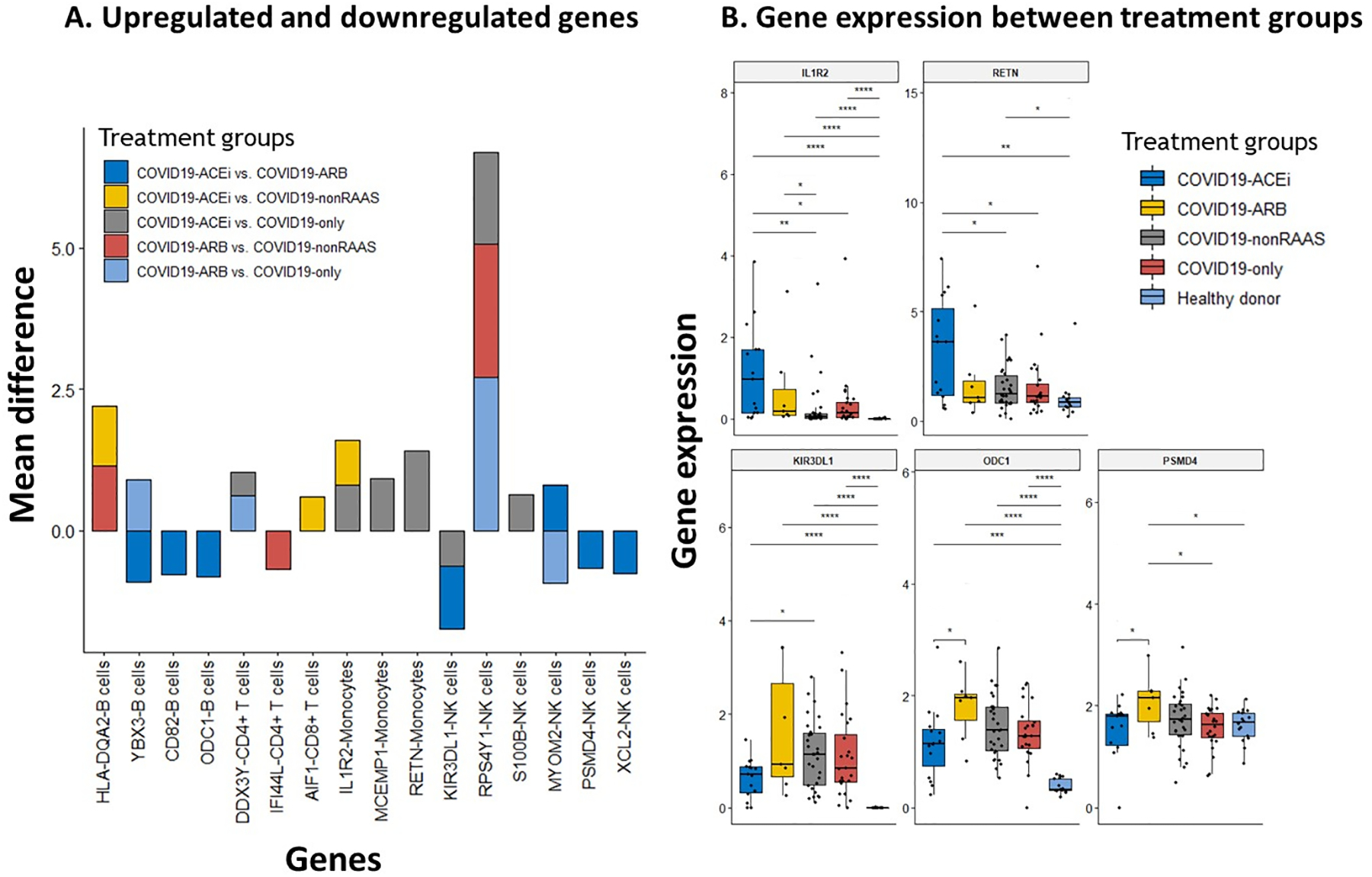
Differential gene expressions across all major immune cell types among hospitalized COVID-19 patients with or without use of antihypertensive drugs. (A) Up-regulated and down-regulated genes, and (B) Gene expression between treatment groups. All these listed genes showed ≥ 2-fold change with significant *P* values. The up- and down-regulation of gene expression was determined based on the mean differences of gene expression between comparative groups. Only genes that were significantly different between treatment groups were presented in Fig. 6B. Statistical significance represented Bonferroni adjusted p-significance in symbols ns: *P* > 0.05, *P ≤ 0.05, **P ≤ 0.01, ***P ≤ 0.001, and ****P ≤ 0.0001. Nonsignificant symbols (ns) were hidden on Fig. 6B.

We next investigated differentially expressed genes between different treatment groups across all major immune cell types. Figure 6A presents up-regulated and down-regulated genes. IL1R2 gene, a decoy receptor [34,35] and an endogenous inhibitor of proinflammatory cytokine interleukin-1 (IL-1), expression in monocytes was significantly (*q* < 0.0001) up-regulated in users of ACEi compared to nonRAAS (4.2 fold change) and COVID19-only (fold change 3.96) (Fig. 6B). RETN gene expression in monocytes was significantly up-regulated (*q* < 0.0002) in COVID19-ACEi compared to COVID-19- only. RETN gene encodes resistin, a proinflammatory cytokine produced in monocytes with a potential immune-suppressive role on neutrophils [36–38]. Past literature also demonstrated the role of ACEi and ARBs in decreasing proinflammatory cytokines and oxidative stress in patients with hypertension [39]. Further, COVID19-ACEi had significantly down-regulated expression of KIR3DL1, a killer immunoglobulin–like receptor expressed by NK cells (*q* < 0.006) compared to COVID19-nonRAAS. ODC1 gene expression on B cells (ornithine decarboxylase), the rate-limiting enzyme for polyamine synthesis [40] was significantly down-regulated in COVID19-ACEi compared to COVID19-ARB (*q* < 0.0001), but this difference was not observed between other treatment groups. PSMD4 gene (proteasome 26S subunit, non-ATPase 4) expression on NK cells was also down-regulated in COVID19-ACEi compared to COVID19-ARB (*q* < 0.0001) (Fig. 6B). Intriguingly, we noticed relatively higher expression levels of anti-inflammatory (IL1R2) [41] and immune suppressive (RETN) genes in MDSC monocytes of hospitalized COVID-19 patients with the use of ACEis; consistent with the immunosuppression function of MDSCs. Though not significant, an elevated expression of IL1R2 trend was also observed in the COVID19-ARB group.

**Fig. 7.**
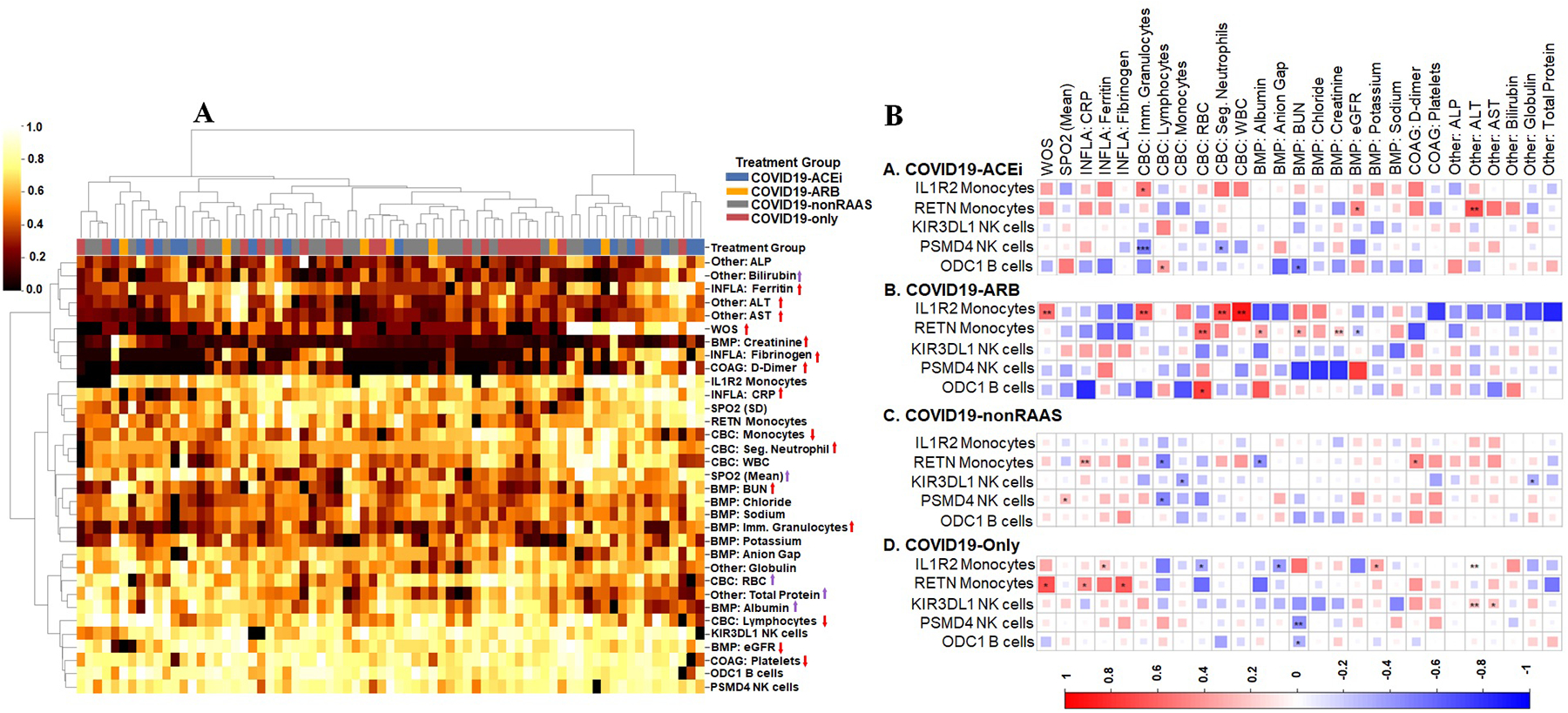
Slight deviations detected between treatment groups. (A) Heatmap illustrates possible clusters. Each column represents 1 INCOV patient corresponding to a treatment group. Rows represent variables that include 27 EHR clinical laboratory markers, 5 genes that showed significant difference, SPO2 (mean, standard deviation [SD]), and disease severity, WOS. Row dendrogram shows the similarity between rows and most similar covariates are placed on branches that are close together. The color bar denotes the distance in (0 to 1, low value and high value). COAG, coagulation labs; BMP, basic metabolic panel; CBC, complete blood counts and rest of labs grouped as Other. SPO2 is a measure of the amount of oxygen-carrying hemoglobin in the blood relative to the amount of hemoglobin not carrying oxygen. Clustering was conducted using the python package, scikit-learn 0.24.2 (sklearn.cluster module). Euclidean distance metric and Ward.D linkage method were used to measure (dis)similarity of covariates and dissimilarity between clusters, respectively. Red arrows associated with each variable (up-faced: higher values and down-faced lower values) are markers of COVID-19 immunopathology [52–54] and SARS-CoV-2-induced immunopathology [52] and disease severity as WOS. The up-faced purple arrows indicate higher values are better. (B) EHR clinical measurements for the treatment groups. Correlation matrix of genes and clinical features from hospitalized treatment groups. The square size corresponds to the absolute value of the Spearman rank correlation coefficient, with red (blue) color indicating a positive (negative) correlation. *FDR < 0.05, **FDR < 0.01, ***FDR < 0.001. Correlation plot was created using R package, corrplot 0.90.

We then pursued hierarchical clustering to examine the similarities between 4 treatment groups by considering 27 EHR clinical laboratory markers, gene expression profiles, SPO2, and disease severity as WOS. No distinctive treatment-based clusters (column-wise) were resolved, but some differences were observed at the variable level (Fig. 7A). Two distinctive row-wise clusters were formed. The genes that are up-regulated in COVID19-ACEi, IL1R2 [41,42] and RETN [36–38] were clustered with markers of COVID-19 immunopathology, SPO2, and 3 other down-regulated genes. The elevated expression of IL1R2 gene in COVID19-ACEi and COVID19-ARB was positively correlated with the infection markers of immune cells. Elevated levels of RETN gene expression in COVID19-ACEi were positively correlated with the estimated glomerular filtration rate, which is used to assess kidney function), and alanine aminotransferase, which can indicate liver dysfunction. Neither lower expression (in COVID19-ACEi) nor higher expression (in COVID19-ARB) of KIR3DL1 was correlated (Fig. 7B).

We next conducted supervised learning to identify variables to classify treatment groups. Both RAAS inhibiting agents (ACEi and ARBs) were grouped as COVID19-RAASi and compared with COVID19-nonRAAS, and COVID19-only (Fig. S4). Anion gap, blood urea nitrogen, creatinine, and globulin were important variables that have higher discriminatory power in classifying COVID19-RAASi from the COVID19-nonRAAS and COVID19-only. Interestingly, the IL1R2 gene is the top ranked variable distinguishing COVID19-RAASi and COVID19-nonRAAS. The extreme gradient boosting classifiers outperformed other models with AUROC for COVID19-RAASi vs. COVID19-nonRAAS (0.76 ± 0.20) and COVID19-RAASi vs. COVID19-only (0.85 ± 0.22) (Table S6). Given the coclustering of elevated expression of anti-inflammatory and immune-suppressor genes with inflammatory markers, we hypothesize that ACE inhibition may moderate inflammation by suppressing proinflammatory cytokines among users of ACEi or ARB [39].

## Discussion

Our study reports that the use of ACEi or ARBs is not associated with the increased risk of receiving IMV, and the 30-d mortality among hospitalized COVID-19 patients was comparable to that of users of other antihypertensive drugs (COVID19-nonRAAS), and those taking no antihypertensive drugs (COVID19-only). Consistent with prior reports [10–18,26,43], our findings reinforce that discontinuation of ACEi and ARBs in patients with indications for RAAS-inhibition agents is not recommended when they become infected with SARS-CoV-2. Although the population of patients taking ACEis may have worse outcomes, this study provides supports that the ACEis themselves are not associated with increased risk. In addition, from gene expression analysis, we observed significantly higher expression of anti-inflammatory and immune suppressive genes in MDSC monocytes among users of COVID19-ACEi compared to COVID19-nonRAAS and COVID19-only.

Compared to angiotensin II receptor type 2 (AT2R), AT1R is highly expressed in immune cells [9]. In contrast to the pathological effector responses of angiotensin II (Ang-II)/AT1R signaling axis in hypertension, AT1R signaling on myeloid and lymphoid cells seems to orchestrate protective immunological responses independent of its role in maintaining blood pressure homeostasis [9]. Consistently, we report and hypothesize the potential role of up-regulated IL1R2 (anti-inflammatory) and RETN (immunosuppressive marker) gene expression in monocytes and prominently in MDSCs of COVID19-ACEi in counterbalancing hyperinflammatory responses. However, these findings require validation in larger sample size. The possible underlying mechanisms of these genes is discussed below.

The increased expression of IL-1 inhibitory receptor 2 (IL1R2) in MDSC monocytes is interesting considering the role of dysregulated monocyte responses and elevated levels of serum inflammatory cytokine, including IL-1β reported in severe COVID-19 patients [42,44,45]. IL-1β can potentiate IL-6 responses that are heavily implicated in excessive inflammation associated with COVID-19 pathogenesis [46]. IL-1β is a potent proinflammatory cytokine whose activity is tightly regulated by various mechanisms that include IL-1 receptor agonists, immunosuppressive cytokines and through decoy receptor, IL-1R2 that exists in membrane bound and soluble forms [47]. Unlike functional signaling that is transduced through IL1R1, IL-1β binding to decoy receptor IL1R2 cannot result in IL-1β-driven inflammatory signals since IL1R2 lacks cytoplasmic signaling domain [48]. Moreover, IL1R2 can also bind and sequester IL-1RAP (accessory protein) required for signal transduction through IL1R1 [49]. Hence, up-regulation of IL1R2 expression in MDSC monocytes of COVID19-ACEi patients may play a key role in attenuating hyperinflammatory responses triggered by CD14 + IL1β monocytes [42].

Resistin (RETN) in humans, demonstrated to be a proinflammatory cytokine, is predominantly secreted by monocytes and macrophages and very little, if any, by mature adipocytes [50]. Interestingly, RETN was shown to exert immunosuppressive effects on neutrophils by reducing neutrophil chemotaxis and oxidative bursts [36–38]. Given the role of neutrophilia and enhanced neutrophil activation in severe COVID-19 outcomes [51] it is interesting to observe enhanced RETN gene expression in monocytes of COVID19-ACEi; whether this enhanced expression of RETN plays an immune-modulatory role on neutrophils in the context of SARS-CoV2 infection remains to be investigated.

Our study has several potential limitations that should be considered in the interpretation of our findings. First, given the retrospective observational study design from EHR, data quality is dependent on how medical information is entered in the system. Secondly, we assumed patient adherence to prescriptions, did not consider the dose of antihypertensive drugs, and did not consider whether the dose of anticoagulant therapy was prophylactic or therapeutic. Third, this study uses hypertension diagnosis to represent a history of high blood pressure but did not include baseline ambulatory blood pressure, which would be an interesting area for future research. Additionally, the PS-matching cohort could have introduced potential selection bias, which we offset by presenting results of KM and Cox regression for both unmatched and PS-matched cohorts.

The results from the gene expression data need to be carefully interpreted because the analysis was based on top 150 genes (selected on the basis of stringent preprocessing procedures), and because the study had a small sample size and unmatched group comparisons due to small size of each of the treatment groups. Hence, future investigations on ACEIs with single cell analysis may benefit from larger cohort of COVID-19 patients and exploration of a larger number of genes.

In summary, results accrued from the retrospective observational data reinforces the continuous use of antihypertensive medications, ACEis, and ARBs when indicated for hypertension, cardiovascular disease, coronary artery disease or chronic kidney disease. Also, this is the first study to report the up-regulation of IL1R2 and RETN genes in monocytes among the users of COVID19-ACEi, which may play a significant role to counterbalance proinflammatory responses.

## Supplementary Material

Supplementary Material Table S1. Categories in the WHO-recommended ordinal scale.
Table S2. SNOMED-CT^1^ codes used for extracting preexisting comorbidities.
Table S3. RxCUI codes used for extracting medications from the EHRs.
Table S4. PS-matched Cohorts^†^.
Table S5. COVID-19 Clinical outcomes by treatment groups (unmatched): score on ordinal scale.
Table S6. Supervised learning models and evaluation metrics.
Table S7. List of Abbreviations.
Fig. S1. Covariate balance before and after PS matching.
Fig. S2. Study Populations and Cohort Selection.
Fig. S3. KM survival curves using unmatched data of hospitalized patients with COVID-19 with or without current use of antihypertensive drugs.
Fig. S4. Classification identified important variables that classify treatment groups.

## Figures and Tables

**Table 1.** Baseline characteristics of COVID-19 patients before propensity score matching^†^

Covariates	COVID19-ACEi	COVID19-ARB	COVID19-nonRAAS	COVID19-only	Total	p value
	count (%)	count (%)	count (%)	count (%)	count (%)	
Total	3342 (100)	2428 (100)	4222 (100)	9798 (100)	19790 (100)	
Age range						<0.001
18–44	212 (6.3)	85 (3.5)	491 (11.6)	2580 (26.3)	3368 (17.0)	
45–54	370 (11.1)	178 (7.3)	499 (11.8)	1660 (16.9)	2707 (13.7)	
55–64	641 (19.2)	413 (17.0)	815 (19.3)	2040 (20.8)	3909 (19.8)	
65–74	936 (28.0)	675 (27.8)	964 (22.8)	1703 (17.4)	4278 (21.7)	
75–84	735 (22.0)	632 (26.0)	788 (18.7)	1091 (11.1)	3246 (16.4)	
85+	448 (13.4)	445 (18.3)	665 (15.8)	724 (7.4)	2282 (11.5)	
Sex						<0.001
Female	1441 (43.1)	1187 (48.9)	2026 (48.0)	4471 (45.6)	9125 (46.1)	
Male	1901 (56.9)	1241 (51.1)	2196 (52.0)	5327 (54.4)	10665 (53.9)	
Ethnicity						<0.001
Hispanic	670 (20.0)	477 (19.6)	934 (22.1)	3022 (30.8)	5103 (25.8)	
Not Hispanic	2633 (78.8)	1925 (79.3)	3227 (76.4)	6476 (66.1)	14261 (72.1)	
Unknown	39 (1.2)	26 (1.1)	61 (1.4)	300 (3.1)	426 (2.1)	
Race						<0.001
AIAN	66 (2.0)	26 (1.1)	68 (1.6)	127 (1.3)	287 (1.4)	
Asian	123 (3.7)	184 (7.6)	184 (4.4)	487 (5.0)	978 (4.9)	
Black	122 (3.7)	133 (5.5)	180 (4.3)	413 (4.2)	848 (4.3)	
NHPI	77 (2.3)	32 (1.3)	50 (1.2)	92 (0.9)	251 (1.3)	
Other/Unknown: Hispanic	438 (13.1)	295 (12.1)	07 (14.4)	1868 (19.1)	3208 (16.2)	
Other/Unknown: Not Hispanic	164 (4.9)	148 (6.1)	239 (5.7)	640 (6.5)	1191 (6.0)	
Other/Unknown, Ethnicity Unknown	39 (1.2)	26 (1.1)	61 (1.4)	300 (3.1)	426 (2.1)	
White	2313 (69.2)	1584 (65.2)	2833 (67.1)	5871 (59.9)	12601 (63.8)	
Preexisting Comorbidities						
Obesity	789 (23.6)	533 (22.0)	708 (16.8)	1202 (12.3)	3232 (16.3)	<0.001
Type 1 DM	53 (1.6)	25 (1.0)	29 (0.7)	47 (0.5)	154 (0.8)	<0.001
Type 2 DM	1327 (39.7)	907 (37.4)	751 (17.8)	1145 (11.7)	4130 (20.9)	<0.001
Hypertension	1381 (41.3)	1039 (42.8)	827 (19.6)	916 (9.3)	4163 (21.0)	<0.001
Atrial fibrillation	581 (17.4)	495 (20.4)	670 (15.9)	553 (5.6)	2299 (11.6)	<0.001
Coronary artery disease (CAD)	610 (18.3)	504 (20.8)	413 (9.8)	319 (3.3)	1846 (9.3)	<0.001
Deep vein thrombosis (DVT)	172 (5.1)	144 (5.9)	247 (5.9)	199 (2.0)	762 (3.8)	<0.001
Myocardial infarction	304 (9.1)	221 (9.1)	223 (5.3)	164 (1.7)	912 (4.6)	<0.001
Heart failure	756 (22.6)	591 (24.3)	518 (12.3)	358 (3.7)	2223 (11.2)	<0.001
Tachycardia	124 (3.7)	65 (2.7)	134 (3.2)	140 (1.4)	463 (2.3)	<0.001
Hypertrophic cardiomyopathy (HCM)	12 (0.4)	7 (0.3)	10 (0.2)	6 (0.1)	35 (0.2)	<0.001
Pulmonary hypertension (PAH)	152 (4.5)	131 (5.4)	133 (3.2)	71 (0.7)	487 (2.5)	<0.001
Angina	138 (4.1)	115 (4.7)	102 (2.4)	53 (0.5)	408 (2.1)	<0.001
Peripheral vascular disease (PVD)	243 (7.3)	155 (6.4)	153 (3.6)	97 (1.0)	648 (3.3)	<0.001
Arteriosclerotic vascular disease (AVD)	738 (22.1)	607 (25.0)	518 (12.3)	423 (4.3)	2286 (11.5)	<0.001
Stroke	258 (7.7)	197 (8.1)	209 (5.0)	163 (1.7)	827 (4.2)	<0.001
Chronic kidney disease (CKD)	725 (21.7)	602 (24.8)	535 (12.7)	426 (4.3)	2288 (11.6)	<0.001
COPD	481 (14.4)	326 (13.4)	499 (11.8)	345 (3.5)	1651 (8.3)	<0.001
Chronic liver disease (CLD)	97 (2.9)	60 (2.5)	125 (3.0)	131 (1.3)	412 (2.1)	<0.001
*Recent anticoagulant use*						
Heparin	3192 (95.5)	2325 (95.8)	4094 (97.0)	2987 (30.4)	12598 (63.7)	<0.001
Other Anticoagulants	984 (29.4)	800 (33.0)	1130 (26.7)	320 (3.2)	3234 (16.3)	<0.001
*Recent antidiabetic use*						
Metformin	1261 (37.7)	744 (30.6)	571 (13.5)	373 (3.8)	2949 (14.9)	<0.001
Non-insulin	942 (28.1)	641 (26.4)	432 (10.2)	179 (3.2)	2194 (11.1)	<0.001
Insulin	2208 (66.0)	1545 (63.6)	2192 (51.9)	905 (9.2)	6850 (34.6)	<0.001
*Recent lipid-lowering drug use*						
Statins	2339 (69.9)	1739 (71.6)	1885 (44.6)	647 (6.6)	6610 (33.4)	<0.001
Non-statins	308 (9.2)	278 (11.4)	247 (5.85)	60 (0.6)	893 (4.5)	<0.001

AIAN: American Indian and Alaska Native, NH/PI: Native Hawaiian and other Pacific Islander, COPD: chronic obstructive pulmonary disease; WOS: WHO Ordinal Scale for Clinical Improvement (Table S1). The p-values were calculated using Pearson’s Chi-squared test for categorical variables and Kruskal-Wallis rank-sum test for continuous variables.

† Figure S1 presents covariate balance after using propensity score (PS) matching algorithm. Percentages given are fractions of the patient group reported in the column.

## Data Availability

Retrospective Observational Study using Electronic Health Records: All clinical logic has been shared. Results have been aggregated and reported within this paper to the extent possible while maintaining privacy from personal health information as required by law. All data are archived within Providence St Joseph Health systems in a HIPAA-secure audited compute environment to facilitate verification of study conclusions. [2] Prospective Observational Study with Deep Immunophenotyping: All data and materials can be accessed from our STAR Methods of our published article, Su et al. [21].
