# Supplementary material for: Angiotensin-Converting Enzyme (ACE) Inhibitors May Moderate COVID-19 Hyperinflammatory Response: An Observational Study with Deep Immunophenotyping
Source: Health Data Sci. 2022 Dec 30;2022:0002. doi: 10.34133/hds.0002 (PMC9934012; doi:10.34133/hds.0002)
Supplement: Supplementary Materials — Table S1. Categories in the WHO-recommended ordinal scale. Table S2. SNOMED-CT 1 codes used for extracting preexisting comorbidities. Table S3. RxCUI codes used for extracting medications from the EHRs. Table S4. PS-matched Cohorts †. Table S5. COVID-19 Clinical outcomes by treatment groups (unmatched): score on ordinal scale. Table S6. Supervised learning models and evaluation metrics. Table S7. List of Abbreviations. Fig. S1. Covariate balance before and after PS matching. Fig. S2. Study Populations and Cohort Selection. Fig. S3. KM survival curves using unmatched data of hospitalized patients with COVID-19 with or without current use of antihypertensive drugs. Fig. S4. Classification identified important variables that classify treatment groups. [file hds.0002.f1.docx]

Supplementary Materials for

**Angiotensin-converting enzyme (ACE) inhibitors may moderate COVID-19 hyperinflammatory response: an observational study with deep immunophenotyping**

Venkata R. Duvvuri^1^, Andrew Baumgartner^1^, Sevda Molani^1^, Patricia V. Hernandez^1^, Dan Yuan^1,2^, Ryan T. Roper^1^, Wanessa Matos^1^, Max Robinson^1^, Yapeng Su^1^,  [NaehaSubramanian](https://www.sciencedirect.com/science/article/pii/S0092867420314446#!)[^1^](https://www.sciencedirect.com/science/article/pii/S0092867420314446#!), Jason D. Goldman^3,4,5^, James R. Heath^1,2^, Jennifer J. Hadlock^1^*****

* Corresponding author: Email: jennifer.hadlock@isbscience.org

**This PDF file includes:**

Tables S1 to S7

Figures S1 to S4

| Table S1. Categories in the WHO-recommended ordinal scale | | |
| --- | --- | --- |
| Patient state | **Descriptor** | **Score** |
| Uninfected | No clinical or virological evidence of infection | 0 |
| Ambulatory | No limitation of activities | 1 |
|  | Limitation of activities | 2 |
| Hospitalized - Mild disease | Hospitalized - no oxygen therapy | 3 |
|  | Oxygen by mask or nasal prongs | 4 |
| Hospitalized - Severe disease | Non-invasive ventilation or high-flow oxygen | 5 |
|  | Intubation and mechanical ventilation | 6 |
|  | Ventilation and additional organ support (vasopressors, renal replacement therapy, ECMO) | 7 |
| Dead | Death | 8 |
| 1. World Health Organization (2020a) WHO R&D Blueprint Novel Coronavirus COVID-19 Therapeutic Trial Synopsis 2. ECMO: Extracorporeal membrane oxygenation | | |

| Table S2. SNOMED-CT^1^ codes used for extracting pre-existing comorbidities | |
| --- | --- |
| Comorbidities | **SNOMED** |
| Angina | 194828000 |
| Arteriosclerotic vascular disease (AVD) | 72092001 |
| Atrial fibrillation | 49436004 |
| Chronic kidney disease (CKD) | 709044004 |
| Chronic liver disease (CLD) | 328383001 |
| Chronic obstructive pulmonary disease (COPD) |  |
| Chronic obstructive lung disease | 13645005 |
| Emphysema | 87433001 |
| Chronic bronchitis | 185086009 |
| Coronary artery disease (CAD) | 53741008, 413844008 |
| Deep vein thrombosis (DVT) | 128053003 |
| Heart failure | 84114007 |
| Hypertension |  |
| Essential hypertension | 59621000 |
| Secondary hypertension | 31992008 |
| Hypertrophic cardiomyopathy (HCM) | 233873004 |
| Myocardial infarction | 22298006 |
| Obesity | 414916001 |
| Peripheral vascular disease (PVD) | 400047006 |
| Pulmonary hypertension (PAH) | 70995007 |
| Stroke | 230690007 |
| Tachycardia | 3424008 |
| Type 1 DM | 46635009 |
| Type 2 DM | 44054006 |
| SNOMED CT: The Systematized Nomenclature of Medicine Clinical Terms  *SNOMED CT© is used with the agreement of SNOMED International*, www.snomed.org | |

| Table S3. RxCUI codes used for extracting medications from the electronic health records | | |
| --- | --- | --- |
| Medication Class | **Name** | **RxCUI** |
| Antihypertensive | ACE inhibitors | 18867, 1998, 21102, 3827, 50166, 60245, 29046, 30131, 54552, 35208, 35296, 36908, 38454, 39990 |
|  | ARB | 1091643, 214354, 83515, 83818, 52175, 321064, 73494, 69749 |
|  | Beta-blockers | 149, 597, 1202, 1520, 19484, 19605, 1817, 2116, 20352, 20498,49737,6185,29518, 6918, 7226, 31555, 7801, 7973, 8332, 8620, 8787, 9947, 37546, 37840,10600, |
|  | Calcium-channel blockers | 17767, 1436, 233603, 3443, 4316, 4327, 4648, 33910, 28382, 135056, 6390, 29275, 39879, 83213, 7396, 7417, 53692, 7426, 7435, 7441, 8050, 11170 |
|  | Diuretics | 644,1808,618970,1982,21914,2603,302285, 298869,62349,  4603,6774, 33770,59743,9997, 10437,358257, 38413,10763 |
|  | Thiazide Diuretics | 1369, 2396,2409,3000,22033,5487,5495,5764,6696,6860, 6916,8565,  10772,11371 |
| Antidiabetics | Insulins and analogue antidiabetics | 253182, 1309342, 86009, 51428, 1670007, 139825, 274783, 400008, 1858994, 1727493 |
|  | Non-insulin ^a^ (Metformin excluded) | 4821, 4815, 4816, 25789, 2404, 10635, 10633, 73044, 274332, 84108, 33738, 16681, 30009, 593411, 857974, 1368001, 1100699, 1368001, 1488564, 1373458, 1545653, 1760, 141626, 1534763, 1551291, 60548, 475968, 1440051, 1991302 |
|  | Metformin | 6809 |
| Lipid-lowering | Statins ^c^ | 36567, 6472, 42463, 41127, 83367, 596723, 301542 |
|  | Non-Statins ^d^ | 2594, 1525, 4719, 8703, 21149, 24609, 1433887, 141626, 2447, 2685, 7393, 104486, 7414, 16817, 3292, 8699, 38248, 69440, 341248, 1367839, 1364479, 1665684, 1659152, 2282403 |
| Anticoagulants | Heparin ^e^ | 5224, 67109, 67108, 67031, 69528, 75960, 78484, 69646, 280611 |
|  | Other anticoagulants ^g^ | 1598, 8130, 11289, 8150, 154, 163426, 50097, 237057, 1037042, 1114195, 1364430, 1599538, 1927851,15202, 60819, 114934, 237057, 321208 |
| RxCUI: RxNorm concept unique identifier, https://mor.nlm.nih.gov/RxNav  ACE inhibitor: Angiotensin-converting enzyme inhibitors  ARB: Angiotensin II receptor blockers  LMWH: Low-molecular-weight heparin  UFH: Unfractionated heparin  ^a^ Non-insulin drugs: Sulfonylureas, Meglitinides, Thiazolidinediones (TZD), Alpha-glucosidase Inhibitors, DPP-4 inhibitors, SGLT2 inhibitors, Cycloset (Dopamine Receptor Agonists), Bile Acid Sequestrants, GLP-1 receptor agonists  ^b^ Metformin: Biguanides  ^c^ Statins: HMG-CoA reductase inhibitors  ^d^ Non-statins: Fibrates, Bile acid sequestrates, Nicotinic acid and derivatives, Other lipid modifying agents  ^e^ Heparin: B01AB Heparin group  ^f^ Vitamin K antagonists, Direct thrombin inhibitors, Direct factor Xa inhibitors | | |

| Table S4. Propensity Score (PS)-matched Cohorts^†^ | | | | | | | | | | | |
| --- | --- | --- | --- | --- | --- | --- | --- | --- | --- | --- | --- |
| Covariates | **COVID19-ACEi vs. COVID19-ARB** | | **COVID19-ACEi vs.**  **COVID19-nonRAAS** | | **COVID19-ACEi vs.**  **COVID19-Only** | | **COVID19-ARB vs.**  **COVID19-nonRAAS** | | **COVID19-ARB vs.**  **COVID19-Only** | |  |
|  | **n=2167** | **n=2167** | **n=3047** | **n=3047** | **n=1085** | **n=1085** | **n=2303** | **n=2303** | **n=835** | **n=835** |  |
| Age in years mean ± SD | 71.21 ± 13.71 | 71.26 ± 13.48 | 69.78 ± 14.26 | 69.55 ± 15.45 | 63.34 ± 15.74 | 63.46 ± 16.38 | 71.7 ± 13.54 | 72.11 ± 13.77 | 66.85 ± 14.39 | 67.68 ± 15.19 |  |
| Sex |  |  |  |  |  |  |  |  |  |  |  |
| Female | 1021 (47.1) | 1035 (47.8) | 1355 (44.5) | 1346 (44.2) | 459 (42.3) | 469 (43.2) | 1123 (48.8) | 1118 (48.5) | 393 (47.1) | 401 (48.0) |  |
| Male | 1146 (52.9) | 1132 (52.2) | 1692 (55.5) | 1701 (55.8) | 626 (57.7) | 616 (56.8) | 1180 (51.2) | 1185 (51.5) | 442 (52.9) | 434 (52.0) |  |
| Ethnicity |  |  |  |  |  |  |  |  |  |  |  |
| Hispanic | 452 (20.9) | 442 (20.4) | 611 (20.1) | 610 (20.0) | 277 (25.5) | 299 (27.6) | 460 (20.0) | 476 (20.7) | 199 (23.8) | 200 (24.0) |  |
| Non-Hispanic | 1693 (78.1) | 1701 (78.5) | 2399 (78.7) | 2402 (78.8) | 791 (72.9) | 771 (71.1) | 1819 (79.0) | 1805 (78.4) | 624 (74.7) | 624 (74.7) |  |
| Unknown | 22 (1.0) | 24 (1.1) | 37 (1.2) | 35 (1.1) | 17 (1.6) | 15 (1.4) | 24 (1.0) | 22 (1.0) | 12 (1.4) | 11 (1.3) |  |
| Reported Race |  |  |  |  |  |  |  |  |  |  |  |
| AIAN | 25 (1.2) | 26 (1.2) | 54 (1.8) | 59 (1.9) | 20 (1.8) | 21 (1.9) | 26 (1.1) | 22 (1.0) | 12 (1.4) | 7 (0.8) |  |
| Asian | 95 (4.4) | 105 (4.8) | 109 (3.6) | 116 (3.8) | 47 (4.3) | 40 (3.7) | 156 (6.8) | 141 (6.1) | 50 (6.0) | 57 (6.8) |  |
| Black | 98 (4.5) | 107 (4.9) | 118 (3.9) | 131 (4.3) | 47 (4.3) | 43 (4.0) | 127 (5.5) | 125 (5.4) | 41 (4.9) | 38 (4.6) |  |
| NHPI | 27 (1.2) | 27 (1.2) | 60 (2.0) | 58 (1.9) | 21 (1.9) | 13 (1.2) | 28 (1.2) | 27 (1.2) | 9 (1.1) | 8 (1.0) |  |
| Other/Unknown  Hispanic | 289 (13.3) | 279 (12.9) | 391 (12.8) | 381 (12.5) | 166 (15.3) | 183 (16.9) | 286 (12.4) | 301 (13.1) | 121 (14.5) | 125 (15.0) |  |
| Other Unknown  Not Hispanic | 117 (5.4) | 115 (5.3) | 149 (4.9) | 159 (5.2) | 54 (5.0) | 49 (4.5) | 133 (5.8) | 143 (6.2) | 49 (5.9) | 53 (6.3) |  |
| Other/Unknown  Ethnicity Unknown | 22 (1.0) | 24 (1.1) | 37 (1.2) | 35 (1.1) | 17 (1.6) | 15 (1.4) | 24 (1.0) | 22 (1.0) | 12 (1.4) | 11 (1.3) |  |
| White | 1494 (68.9) | 1484 (68.5) | 2129 (69.9) | 2108 (69.2) | 713 (65.7) | 721 (66.5) | 1523 (66.1) | 1522 (66.1) | 541 (64.8) | 536 (64.2) |  |
| Preexisting Comorbidities |  |  |  |  |  |  |  |  |  |  |  |
| Obesity | 480 (22.2) | 486 (22.4) | 697 (22.9) | 680 (22.3) | 202 (18.6) | 173 (15.9) | 498 (21.6) | 477 (20.7) | 143 (17.1) | 125 (15.0) |  |
| Type 1 DM | 26 (1.2) | 25 (1.2) | 38 (1.2) | 37 (1.2) | 7 (0.6) | 12 (1.1) | 25 (1.1) | 22 (1.0) | 6 (0.7) | 5 (0.6) |  |
| Type 2 DM | 796 (36.7) | 809 (37.3) | 1125 (36.9) | 1121 (36.8) | 238 (21.9) | 242 (22.3) | 835 (36.3) | 794 (34.5) | 195 (23.4) | 188 (22.5) |  |
| Hypertension | 937 (43.2) | 935 (43.1) | 1203 (39.5) | 1248 (41.0) | 177 (16.3) | 164 (15.1) | 971 (42.2) | 995 (43.2) | 173 (20.7) | 140 (16.8) |  |
| Atrial fibrillation | 427 (19.7) | 422 (19.5) | 531 (17.4) | 520 (17.1) | 83 (7.6) | 69 (6.4) | 462 (20.1) | 420 (18.2) | 60 (7.2) | 63 (7.5) |  |
| Coronary artery disease (CAD) | 439 (20.3) | 442 (20.4) | 541 (17.8) | 546 (17.9) | 65 (6.0) | 60 (5.5) | 467 (20.3) | 460 (20.0) | 53 (6.3) | 49 (5.9) |  |
| Deep vein thrombosis (DVT) | 124 (5.7) | 128 (5.9) | 152 (5.0) | 148 (4.9) | 41 (3.8) | 38 (3.5) | 131 (5.7) | 133 (5.8) | 36 (4.3) | 38 (4.6) |  |
| Myocardial infarction | 197 (9.1) | 199 (9.2) | 269 (8.8) | 258 (8.5) | 34 (3.1) | 29 (2.7) | 206 (8.9) | 192 (8.3) | 27 (3.2) | 26 (3.1) |  |
| Heart failure | 506 (23.4) | 525 (24.2) | 676 (22.2) | 689 (22.6) | 77 (7.1) | 61 (5.6) | 544 (23.6) | 547 (23.8) | 64 (7.7) | 48 (5.7) |  |
| Tachycardia | 58 (2.7) | 60 (2.8) | 107 (3.5) | 91 (3.0) | 24 (2.2) | 26 (2.4) | 62 (2.7) | 59 (2.6) | 20 (2.4) | 14 (1.7) |  |
| Hypertrophic cardiomyopathy (HCM) | 7 (0.3) | 7 (0.3) | 11 (0.4) | 10 (0.3) | 0 (0.0) | 1 (0.1) | 7 (0.3) | 7 (0.3) | 1 (0.1) | 0 (0.0) |  |
| Pulmonary hypertension (PAH) | 113 (5.2) | 106 (4.9) | 136 (4.5) | 139 (4.6) | 17 (1.6) | 15 (1.4) | 119 (5.2) | 120 (5.2) | 18 (2.2) | 13 (1.6) |  |
| Angina | 105 (4.8) | 109 (5.0) | 124 (4.1) | 120 (3.9) | 14 (1.3) | 10 (0.9) | 107 (4.6) | 101 (4.4) | 13 (1.6) | 14 (1.7) |  |
| Peripheral vascular disease (PVD) | 138 (6.4) | 146 (6.7) | 204 (6.7) | 220 (7.2) | 27 (2.5) | 22 (2.0) | 144 (6.3) | 139 (6.0) | 23 (2.8) | 16 (1.9) |  |
| Arteriosclerotic vascular disease (AVD) | 538 (24.8) | 527 (24.3) | 660 (21.7) | 671 (22.0) | 86 (7.9) | 88 (8.1) | 566 (24.6) | 558 (24.2) | 73 (8.7) | 60 (7.2) |  |
| Stroke | 189 (8.7) | 177 (8.2) | 233 (7.6) | 226 (7.4) | 37 (3.4) | 31 (2.9) | 190 (8.3) | 180 (7.8) | 29 (3.5) | 27 (3.2) |  |
| Chronic kidney disease (CKD) | 524 (24.2) | 523 (24.1) | 643 (21.1) | 640 (21.0) | 92 (8.5) | 82 (7.6) | 558 (24.2) | 535 (23.2) | 99 (11.9) | 78 (9.3) |  |
| Chronic obstructive pulmonary disease (COPD) | 286 (13.2) | 300 (13.8) | 439 (14.4) | 434 (14.2) | 98 (9.0) | 92 (8.5) | 314 (13.6) | 310 (13.5) | 71 (8.5) | 64 (7.7) |  |
| Chronic liver disease (CLD) | 49 (2.3) | 56 (2.6) | 88 (2.9) | 81 (2.7) | 22 (2.0) | 20 (1.8) | 59 (2.6) | 49 (2.1) | 16 (1.9) | 18 (2.2) |  |
| Current Medication History |  |  |  |  |  |  |  |  |  |  |  |
| *Anticoagulants* |  |  |  |  |  |  |  |  |  |  |  |
| Heparin | 2072 (95.6) | 2068 (95.4) | 2917 (95.7) | 2915 (95.7) | 983 (90.6) | 997 (91.9) | 2203 (95.7) | 2218 (96.3) | 759 (90.9) | 773 (92.6) |  |
| Other Anticoagulants | 697 (32.2) | 695 (32.1) | 892 (29.3) | 891 (29.2) | 912 (84.1) | 918 (84.6) | 749 (32.5) | 705 (30.6) | 140 (16.8) | 144 (17.2) |  |
| *Antidiabetics* |  |  |  |  |  |  |  |  |  |  |  |
| Metformin | 680 (31.4) | 687 (31.7) | 1077 (35.3) | 827 (27.1) | 254 (23.4) | 254 (23.4) | 686 (29.8) | 626 (27.2) | 653 (78.2) | 663 (79.4) |  |
| Non-insulin | 569 (26.3) | 570 (26.3) | 800 (26.3) | 651 (21.4) | 138 (12.7) | 146 (13.5) | 586 (25.4) | 481 (20.9) | 113 (13.5) | 114 (13.7) |  |
| Insulin | 1368 (63.1) | 1368 (63.1) | 1950 (64.0) | 1856 (60.9) | 518 (47.7) | 520 (47.9) | 1442 (62.6) | 1388 (60.3) | 397 (47.5) | 386 (46.2) |  |
| *Lipid-lowering drugs* |  |  |  |  |  |  |  |  |  |  |  |
| Statins | 1533 (70.7) | 1549 (71.5) | 2100 (68.9) | 1893 (62.1) | 478 (44.1) | 472 (43.5) | 1637 (71.1) | 1446 (62.8) | 563 (64.7) | 555 (63.8) |  |
| Non-statins | 231 (10.7) | 229 (10.6) | 265 (8.7) | 294 (9.6) | 35 (3.2) | 38 (3.5) | 258 (11.2) | 191 (8.3) | 41 (4.7) | 37 (4.3) |  |
| Outcome: Score on Ordinal Scale |  |  |  |  |  |  |  |  |  |  |  |
| WOS 6 or 7 | 109 (5.0) | 101 (4.7) | 160 (5.3) | 184 (6.0) | 56 (5.2) | 26 (2.4) | 108 (4.7) | 142 (6.2) | 386 (46.2) | 397 (47.5) |  |
| WOS 8 | 237 (10.9) | 235 (10.8) | 288 (9.5) | 451 (14.8) | 93 (8.6) | 88 (8.1) | 268 (11.6) | 344 (14.9) | 44 (5.3) | 44 (5.3) |  |
| AIAN: American Indian and Alaska Native, NH/PI: Native Hawaiian and other Pacific Islander, other: unknown race, Other Hispanic: unknown race, Hispanic ethnicity; and Other non-Hispanic: unknown race, non-Hispanic ethnicity, COPD: chronic obstructive pulmonary disease; WOS: WHO Ordinal Scale for Clinical Improvement (Table S1). The p-values were calculated using Pearson’s Chi-squared test for categorical variables and Kruskal-Wallis rank-sum test for continuous variables. † Figure S1 presents covariate balance after using propensity score (PS) matching algorithm. Percentages given are fractions of the patient group reported in the column | | | | | | | | | | | |

| **Table S5. COVID-19 Clinical outcomes by treatment groups (unmatched): score on ordinal scale** | | | | | | | |
| --- | --- | --- | --- | --- | --- | --- | --- |
| **Score** | **COVID19-Only** | **Total anti-hypertensive** | **χ^2^**  **p value** | **COVID19- nonRAAS** | **COVID19-ARB** | **COVID19-ACEi** | **χ^2^**  **p value** |
| 3, 4. Hospitalized Mild | 6721 (68.6%) | 6117 (61.2%) | 228.1  <0.001 | 2329 (55.2%) | 1573 (64.8%) | 2215 (66.3%) | 183.9  <0.001 |
| 5. Hospitalized Severe, receiving noninvasive ventilation or high-flow oxygen devices | 1205 (12.3%) | 1699 (17.2%) |  | 709 (16.8%) | 417 (17.2%) | 573 (17.1%) |  |
| 6, 7. Hospitalized Severe, receiving invasive mechanical ventilation | 402 (4.1%) | 771 (7.7%) |  | 399 (9.5%) | 150 (6.2%) | 222 (6.6%) |  |
| 8. Death | 1470 (15%) | 1405 (14.1%) |  | 785 (18.6%) | 288 (11.9%) | 332 (9.9%) |  |
| Total | 9798 | 9992 |  | 4222 | 2428 | 3342 |  |

| Table S6. Supervised learning models and evaluation metrics | | | | | | |
| --- | --- | --- | --- | --- | --- | --- |
| Compare | Supervised learning  Model | Accuracy | Positive Predictive Value (PPV) | Sensitivity (TPR) | F1 score | AUROC |
| COVID19-RAASi vs. COVID19-nonRAAS | LR | 0.60 ± 0.18 | 0.50 ± 0.26 | 0.57 ± 0.18 | 0.55 ± 0.20 | 0.63 ± 0.31 |
|  | AdaBoost | 0.66 ± 0.12 | 0.64 ± 0.25 | 0.63 ± 0.13 | 0.61 ± 0.15 | 0.66 ± 0.21 |
|  | RF | 0.66 ± 0.17 | 0.64 ± 0.25 | 0.64 ± 0.18 | 0.62 ± 0.19 | 0.76 ± 0.21 |
|  | GB | 0.64 ± 0.26 | 0.61 ± 0.31 | 0.63 ± 0.26 | 0.62 ± 0.28 | 0.67 ± 0.26 |
|  | XGBoost* | 0.76 ± 0.12 | 0.81 ± 0.12 | 0.75 ± 0.11 | 0.75 ± 0.12 | 0.76 ± 0.20 |
| COVID19-RAASi vs. COVID19-only | LR | 0.76 ± 0.24 | 0.79 ± 0.25 | 0.76 ± 0.23 | 0.74 ± 0.25 | 0.88 ± 0.16 |
|  | AdaBoost | 0.70 ± 0.12 | 0.69 ± 0.30 | 0.70 ± 0.24 | 0.67 ± 0.27 | 0.80 ± 0.24 |
|  | RF | 0.76 ± 0.17 | 0.77 ± 0.26 | 0.76 ± 0.24 | 0.75 ± 0.25 | 0.80 ± 0.26 |
|  | GB | 0.71 ± 0.26 | 0.69 ± 0.33 | 0.71 ± 0.27 | 0.68 ± 0.30 | 0.78 ± 0.26 |
|  | XGBoost* | 0.78 ± 0.12 | 0.77 ± 0.29 | 0.79 ± 0.23 | 0.76 ± 0.27 | 0.85 ± 0.22 |
| LR: Logistic Regression; AdaBoost: Adaptive Boosting; RF: Random Forest; GB: Gradient Boosting; XGBoost: eXtreme Gradient Boosting and SVM: Support Vector Machine  Accuracy = (TP + TN)/ (TP + FP + TN + FN),  Positive Predictive Value (PPV or Precision) = (TP)/(TP+FP),  Sensitivity (TPR or Recall) = (TP)/(TP+FN),  F1-score = 2 * (PPV * TPR / PPV + TPR)  TP = True Positive; FP = False Positive; TN = True Negative; FN = False Negative; TPR = True Positive Rate; PPV = Positive Predictive Value, AUROC: Area Under Curve of Receiver Operating Characteristic Curve  * XGBoost outperformed all other models | | | | | | |

| Table S7. List of Abbreviations | | |
| --- | --- | --- |
| Covariate related to | **Abbreviations** | **Full terms** |
| Race | AIAN | American Indian and Alaska Native |
|  | NHPI | Native Hawaiian and other Pacific Islander |
| Preexisting conditions | CAD | Coronary artery disease |
|  | CKD | Chronic kidney disease |
|  | CLD | Chronic liver disease |
|  | COPD | Chronic obstructive pulmonary disease |
|  | DVT | Deep vein thrombosis |
|  | HCM | Hypertrophic cardiomyopathy |
|  | HF | Heart failure |
|  | HTN | Hypertension |
|  | IMIDs | Immune-mediated inflammatory diseases |
|  | MI | Myocardial infarction |
|  | PAH | Pulmonary hypertension |
|  | Type 1 DM | Type 1 diabetes mellitus |
|  | Type 2 DM | Type 12diabetes mellitus |
| Disease severity | WOS | WHO Ordinal Scale for Clinical Improvement |
| Treatment cohorts | COVID19-ACEi | Hospitalized COVID-19 patients who are current users of ACE inhibitors |
|  | COVID19-ARB | Hospitalized COVID-19 patients who are current users of ARB drug |
|  | COVID19-nonRAAS | Hospitalized COVID-19 patients who are current users of nonRAAS drugs such as beta-blockers, calcium-channel blockers and diuretics |
|  | COVID19-Only | Hospitalized COVID-19 patients who are not on any antihypertensive medications |

**
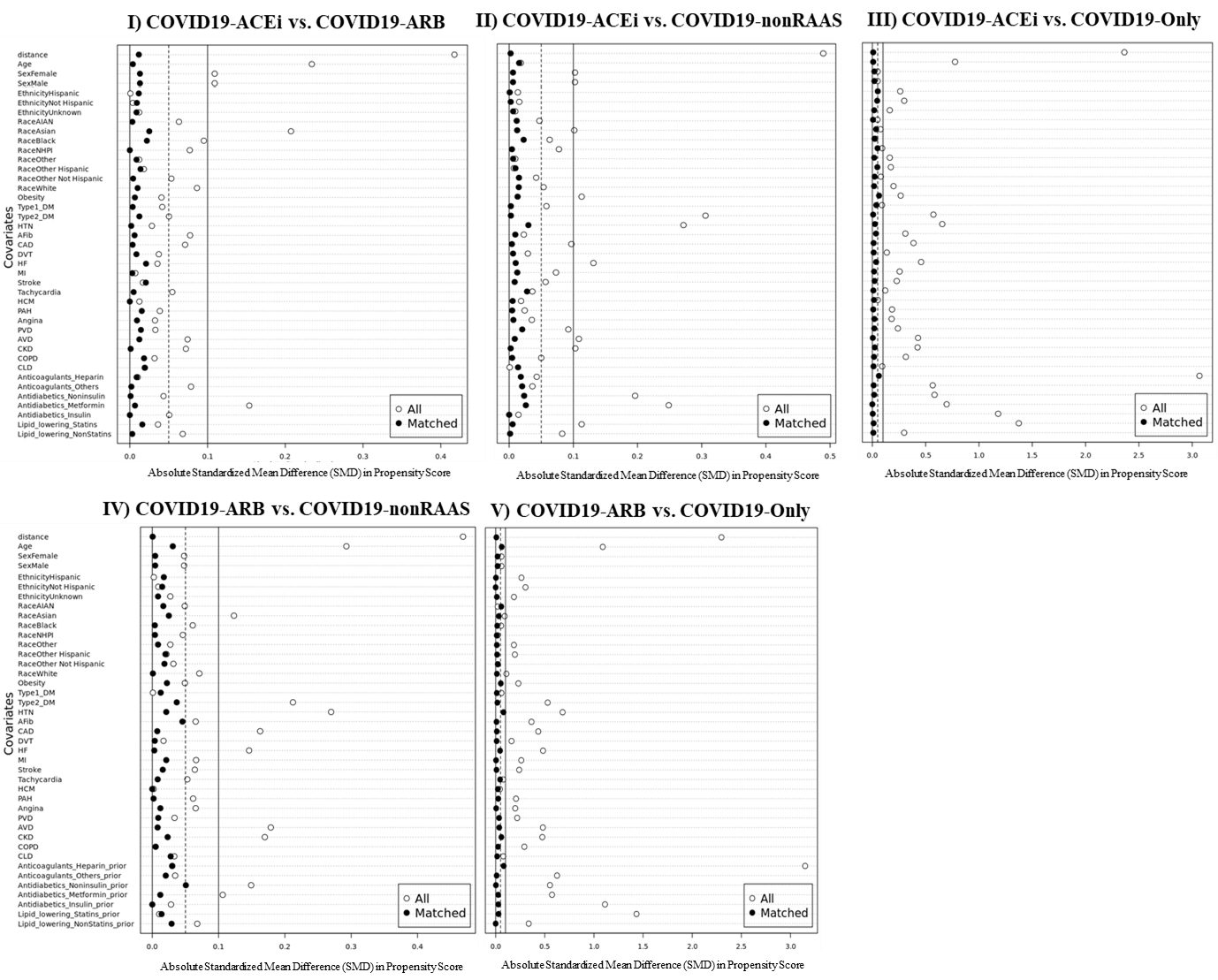
**

**Fig. S1.** **Covariate balance before and after propensity score (PS) matching.**

I) COVID19-ACEi vs. COVID19-ARB; II) COVID19-ACEi vs. COVID19-nonRAAS; III) COVID19-ACEi vs. COVID19-Only; IV) COVID19-ARB vs. COVID19-nonRAAS and V) COVID19-ARB vs. COVID19-Only. The absolute standardized mean difference (SMD) <= 0.1 is considered as an indicator of successful balancing between two groups. White dots indicate (SMD) before PS-matching and solid black dots indicate SMD after PS adjustment. Abbreviations are available in Table S7.

**
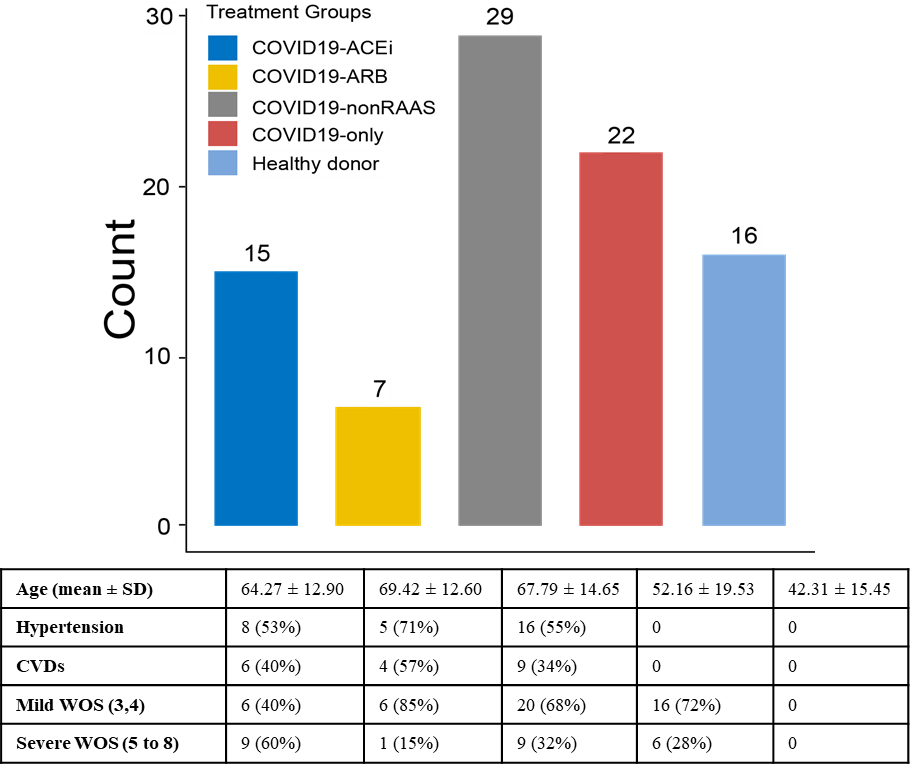
**

**Fig S2.** **Study Populations and Cohort Selection.** Four treatment groups were selected from COVID-19 cases from the prospective observational ISB-Swedish INCOV study (*21*). Healthy controls were the same as those used for analysis of the INCOV study. ACEi: angiotensin-converting enzyme inhibitors. ARB: angiotensin receptor blocker. CVDs: cardiovascular diseases. RAAS: Renin-angiotensin-aldosterone system. WOS: WHO Ordinal Scale for Clinical Improvement (Table S1).

**
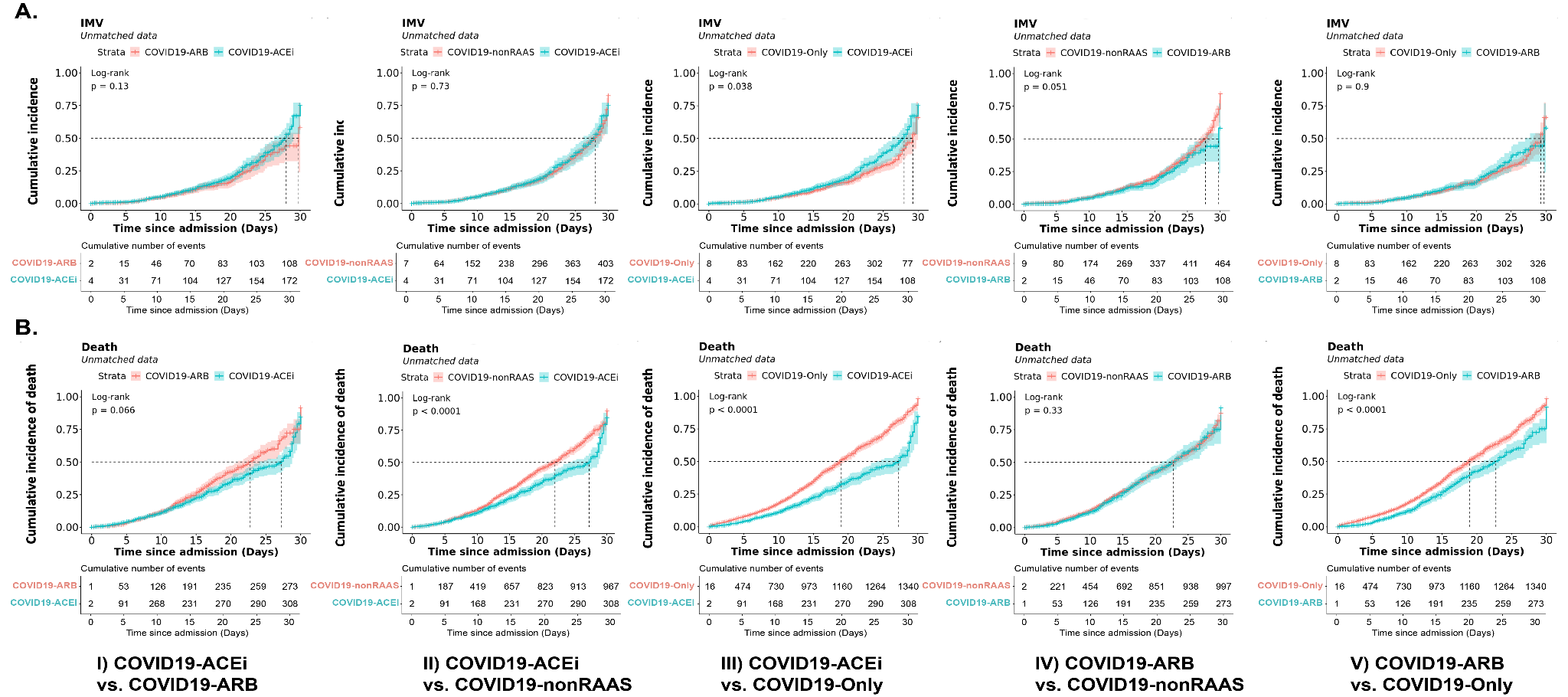
**

**Fig. S3. Kaplan-Meier (KM) survival curves using unmatched data of hospitalized patients with COVID-19 with or without current use of antihypertensive drugs.**

(A) Survival curves for receiving invasive mechanical ventilation (IMV) or ECMO (WOS, 6, 7). (B) Survival curves for mortality (WOS, 8). Survival analysis was conducted using five unmatched cohorts: COVID19-ACEi vs. COVID19-ARB; COVID19-ACEi vs. COVID19-nonRAAS; COVID19-ACEi vs. COVID19-Only; COVID19-ARB vs. COVID19-nonRAAS and COVID19-ARB vs. COVID19-Only. Log-rank P-values are for the difference between treatments where p < 0.05 considered to be significantly different. Numbers provided indicate the cumulative number of events in each cohort. Table 1 presents characteristics of unmatched cohorts

**
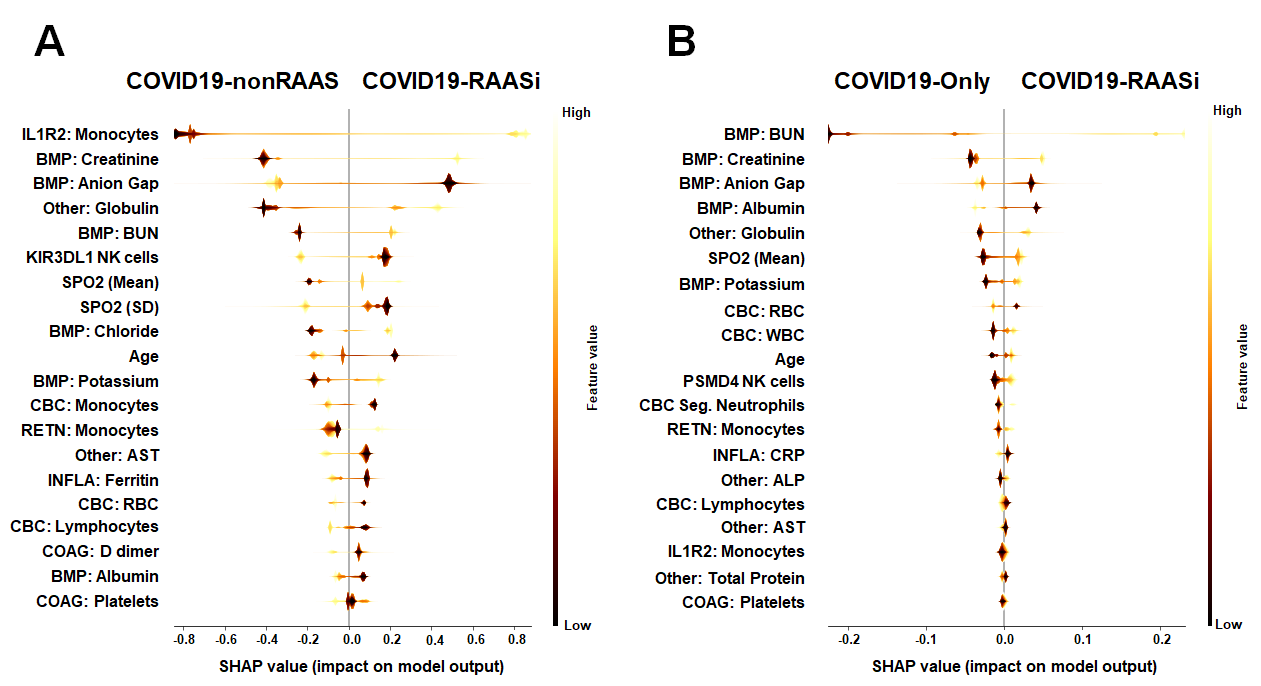
**

**Fig. S4.** **Classification identified important variables that classify treatment groups.** **(A)** SHapley Additive exPlanations (SHAP) layered violin plot presents importance of each variable and its relationship in classifying COVID19-RAASi and COVID19-nonRAAS groups. (B) SHAP (*60*) layered violin plot presents the importance for each variable and its relationship in classifying COVID19-RAASi and COVID19-Only groups. In (B), ACEi and ARB were merged as one group, RAASi (renin-angiotensin-aldosterone system inhibitors). *Understand SHAP plots*: Variables on the y-axis are in order of importance from top to bottom. SHAP values on x-axis indicate how much change in log-odds towards classification. *Interpret SHAP plot*: High IL1R2 Monocytes and low anion gap are associated with COVID19-RAASi group when compared to COVID19-nonRAAS (A) and High blood urea nitrogen (BUN) and low anion gap are associated with COVID19-RAASi group when compared to COVID19-Only (B). SHAP values represent the marginal contribution of each feature value to a given model.
